# Development and comparison of cell-free protein synthesis systems derived from typical bacterial chassis

**DOI:** 10.1186/s40643-021-00413-2

**Published:** 2021-07-06

**Authors:** Liyuan Zhang, Xiaomei Lin, Ting Wang, Wei Guo, Yuan Lu

**Affiliations:** 1 Department of Ecology, Shenyang Agricultural University, Shenyang, 110866 Liaoning Province China; 2 Key Laboratory of Industrial Biocatalysis, Ministry of Education, Department of Chemical Engineering, Tsinghua University, Beijing, 100084 China

**Keywords:** *Escherichia coli*, *Bacillus subtilis*, *Corynebacterium glutamate*, *Vibrio natriegens*, Cell-free protein synthesis system, SARS-CoV-2 RBD protein expression

## Abstract

Cell-free protein synthesis (CFPS) systems have become an ideal choice for pathway prototyping, protein production, and biosensing, due to their high controllability, tolerance, stability, and ability to produce proteins in a short time. At present, the widely used CFPS systems are mainly based on *Escherichia coli* strain. *Bacillus subtilis*, *Corynebacterium glutamate,* and *Vibrio natriegens* are potential chassis cells for many biotechnological applications with their respective characteristics. Therefore, to expand the platform of the CFPS systems and options for protein production, four prokaryotes, *E. coli*, *B. subtilis*, *C. glutamate,* and *V. natriegens* were selected as host organisms to construct the CFPS systems and be compared. Moreover, the process parameters of the CFPS system were optimized, including the codon usage, plasmid synthesis competent cell selection, plasmid concentration, ribosomal binding site (RBS), and CFPS system reagent components. By optimizing and comparing the main influencing factors of different CFPS systems, the systems can be optimized directly for the most influential factors to further improve the protein yield of the systems. In addition, to demonstrate the applicability of the CFPS systems, it was proved that the four CFPS systems all had the potential to produce therapeutic proteins, and they could produce the receptor-binding domain (RBD) protein of SARS-CoV-2 with functional activity. They not only could expand the potential options for in vitro protein production, but also could increase the application range of the system by expanding the cell-free protein synthesis platform.

**Supplementary Information:**

The online version contains supplementary material available at 10.1186/s40643-021-00413-2.

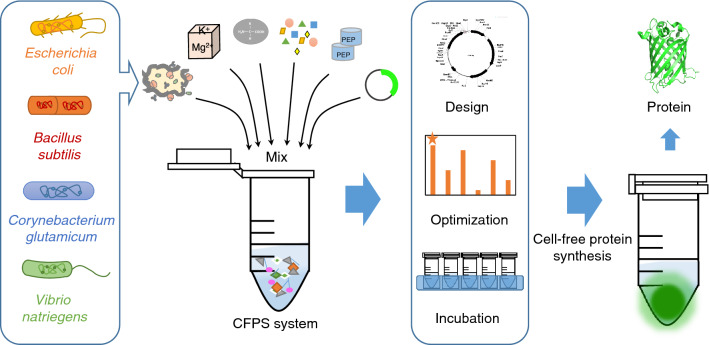

## Introduction

Cell-free protein synthesis (CFPS) is a way for rapid protein synthesis in vitro (Carlson et al. 2012; Gregorio et al. 2019). Extract-based CFPS systems as tools have promoted the development of basic biology and applied biology. They provide some unique advantages for the production and application of proteins. For example, the open environment of the reaction allows users to directly add or synthesize new ingredients at precise concentrations, allowing them to be designed, tested and optimized for different products in a faster, more convenient, and more controlled manner (Sullivan et al. 2016). Higher tolerance facilitates the production of toxic protein products. In addition, CFPS systems can be stored in lyophilized form for up to 1 year, demonstrating greater stability (Smith et al. 2014). These advantages make the CFPS systems an ideal choice for path design, protein production, and personalized medicine (Hodgman and Jewett 2012; Kelwick et al. 2014; Lu 2017). Therefore, they are increasingly used in the production of complex protein products with low expression rates, aggregation, toxicity, and poor solubility in vivo (Schoborg et al. 2018). CFPS systems also have broad application prospects and have been used in the design of rapid prototyping of DNA regulatory elements, logic systems (Chappell et al. 2013; Karim and Jewett 2016; Stech and Kubick 2015), and biosensor devices (Lin et al. 2020a, b; Pardee et al. 2016; Zhang et al. 2020, 2019).

In recent years, taking advantage of the diversity of cell biosynthesis has led to an increase in the number of host species used for different CFPS systems (Zemella et al. 2015). In theory, any organism could be used as a basis for CFPS. CFPS systems are mainly divided into eukaryotic and prokaryotic systems. Yeast, wheat germ, rabbit reticulocyte and insect cell are commonly used as eukaryotic hosts for cell-free systems. Eukaryotic cells, as safe host cells, have complex post-translational modification functions and can produce more complex proteins. However, the high cost of cultivating eukaryotic cells, the more complex process, and the low protein yield in batch reaction make people more inclined to study the CFPS system of prokaryotic host cells (Adiga et al. 2018). Compared with the eukaryotic system, the prokaryotic system has more convenient extraction solution preparation, higher protein yield, lower downstream processing requirements, and lower cost. At present, *Escherichia coli* is the most popular and widely used prokaryotic CFPS system (Failmezger et al. 2017). In addition, cell-free systems of prokaryotic hosts with different model strains such as *Vibrio* (Des Soye et al. 2018; Li et al. 2017; Moore et al. 2017; Wiegand et al. 2018), *Pseudomonas* (Wang et al. 2018) and *Bacillus* (Kelwick et al. 2016; Moore et al. 2018) have also begun to develop in recent years. However, these studies only established relevant systems, but they lacked comparative analysis among different CFPS systems. Therefore, in-depth exploration and comparative analysis should be carried out so as to guide the application of the systems better.

The development of CFPS needs to focus on common and valuable chassis cells, such as *Escherichia coli*, *Bacillus subtilis*, *Corynebacterium glutamicum* and *Vibrio natriegens*. *E. coli*, *B. subtilis*, *C. glutamicum* and *V. natriegens* are reliable and powerful base microorganisms for laboratory research and industrial production. They have different characteristics and can be the focus of the CFPS platform. *E. coli*, as the preferred host cell for the present CFPS systems, has obvious advantages, such as the simple operation of culture conditions and cell lysis methods, the maximum protein yield with several milligrams/ml, and the low cost of cell culture (Failmezger et al. 2017). *B. subtilis* has no obvious codon preference, can avoid codon optimization, and is widely used in industrial protein production (Guan et al. 2016; Jeong et al. 2014; Sarah et al. 2016). *C. glutamicum* has minimal protease activity, which gives this strain a strong potential to express protease-sensitive proteins (Smith et al. 2010; Sun et al. 2016). In addition, both *B. subtilis* and *C. glutamicum* belong to Gram-positive strains and are non-pathogenic microorganisms with less endotoxicity, so they can be safely used in the production of food and pharmaceutical proteins. The doubling time of *V. natriegens* is twice as fast as that of *E. coli*, and it produces a large number of ribosomes that support its robust transcription system of rapid growth, giving *V. natriegens* the potential to achieve excellent productivity in the field of high levels of protein expression (Becker et al. 2019; Dalia et al. 2017; Des Soye et al. 2018; Fernández-Llamosas et al. 2017; Tschirhart et al. 2019; Weinstock et al. 2016; Wiegand et al. 2018). The foregoing features give people new interest in the development and use of CFPS systems based on these strains. Among them, the CFPS systems of *E. coli* are more comprehensive. These cell-free systems have been continuously improved and optimized through various researches, resulting in a high-yield system, and a wide range of applications, including the production of applied proteins and commercial small molecule products, and the design of cell-free biosensors. However, there are few relevant studies on the three CFPS systems of *B. subtilis*, *C. glutamicum* and *V. natriegens*. These three systems should be further studied and optimized, and different CFPS systems should be compared and analyzed. In this way, the advantages and important influencing factors of different systems can be obtained. Through targeted optimization, the efficiency and expression level of the system can be improved to assist further application to select a more effective CFPS system.

**Fig. 1:**
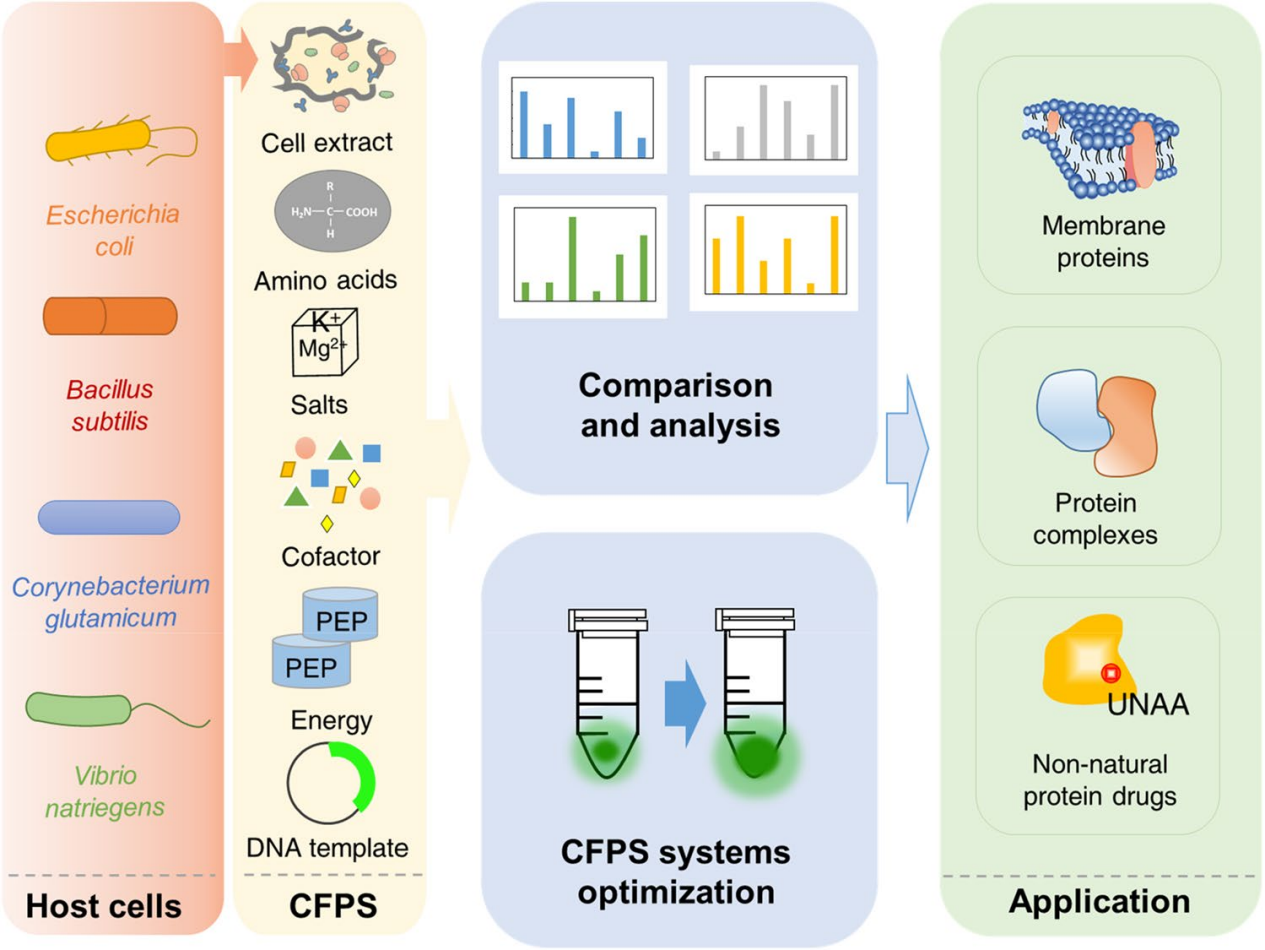
The CFPS system as a platform for basic scientific research, preliminary screening and related applications

Here, this study aims to build a robust and productive CFPS platform based on *E. coli*, *B. subtilis*, *C. glutamicum* and *V. natriegens* (Fig. 1). Then the protein synthesis yield of these systems was improved by systematically optimizing the process parameters. Specifically, the effects of DNA template (including codons, competent cells used for plasmid extraction, plasmid concentration, and RBS) and system reagent components (including Mg^2+^, PEP, NTPs, amino acids, oxidized reductant, and PEG8000) on sfGFP (superfolder green fluorescent protein) synthesis yield were evaluated. Furthermore, the differences and main influencing factors of different CFPS systems were compared and analyzed. Then, to demonstrate the applicability of the CFPS systems, the four CFPS systems were used to express the therapeutic SARS-CoV-2 RBD protein and to examine its activity characterization. It is hoped that by developing the CFPS system in different host cells, the platform range and potential options for in vitro protein production can be expanded, and a variety of highly expressed structural and functional proteins can be produced for applications in pharmaceutical proteins and other fields.

## Results and discussion

### Codon optimization

**Fig. 2:**
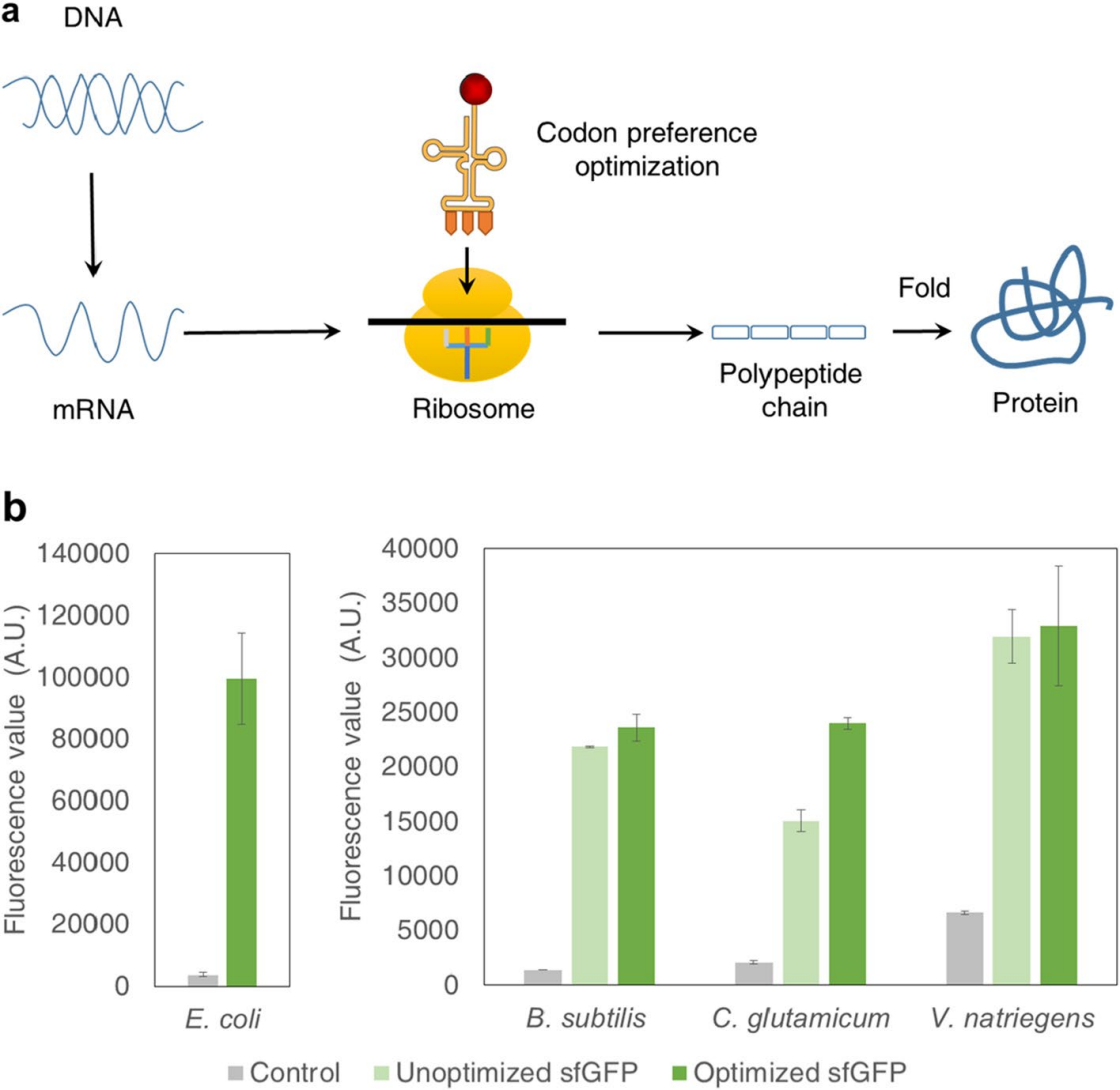
Effect of codon optimization on protein expression of *E. coli*, *B. subtilis*, *C. glutamicum* and *V. natriegens* CFPS systems. **a** Diagram of protein expression process. **b** sfGFP protein synthesis level after codon optimization of *E. coli*, *B. subtilis*, *C. glutamicum* and *V. natriegens* CFPS systems. The mean and standard deviations are shown (N = 3). (The control is the background value of the systems.)

Every organism used for protein expression exhibits some degree of difference or preference in codon utilization. To make use of preferred codons, avoid low-utilization or rare codons, and enable the protein production system to carry out high level expression, the codons of *B. subtilis, C. glutamicum* and *V. natriegens* plasmids were optimized. Codon optimization for *B. subtilis, C. glutamicum* and *V. natriegens* plasmids was based on *E. coli* plasmids. According to the experimental data (Fig. 2), the protein synthesis level of the *B. subtilis* and *V. natriegens* CFPS systems was only slightly increased after codon optimization. The codon optimization had no significant effect on the protein synthesis of these systems, possibly because the bacteria itself had no obvious codon preference. However, for *C. glutamate* CFPS system, codon optimization could significantly increase the protein synthesis level of the system by nearly 30–40%. Therefore, when *B. subtilis* system and *V. natriegens* system were used for the expression of functional proteins in the future, the step of codon optimization could be omitted. In the *C. glutamicum* system, the protein expression could be improved through the codon optimization. In addition, from the protein synthesis level of these systems, the initial protein synthesis level of the *B. subtilis, C. glutamicum* and *V. natriegens* CFPS systems were much lower than that of the *E. coli* CFPS system.

### Optimization of plasmid source and plasmid concentration

**Fig. 3:**
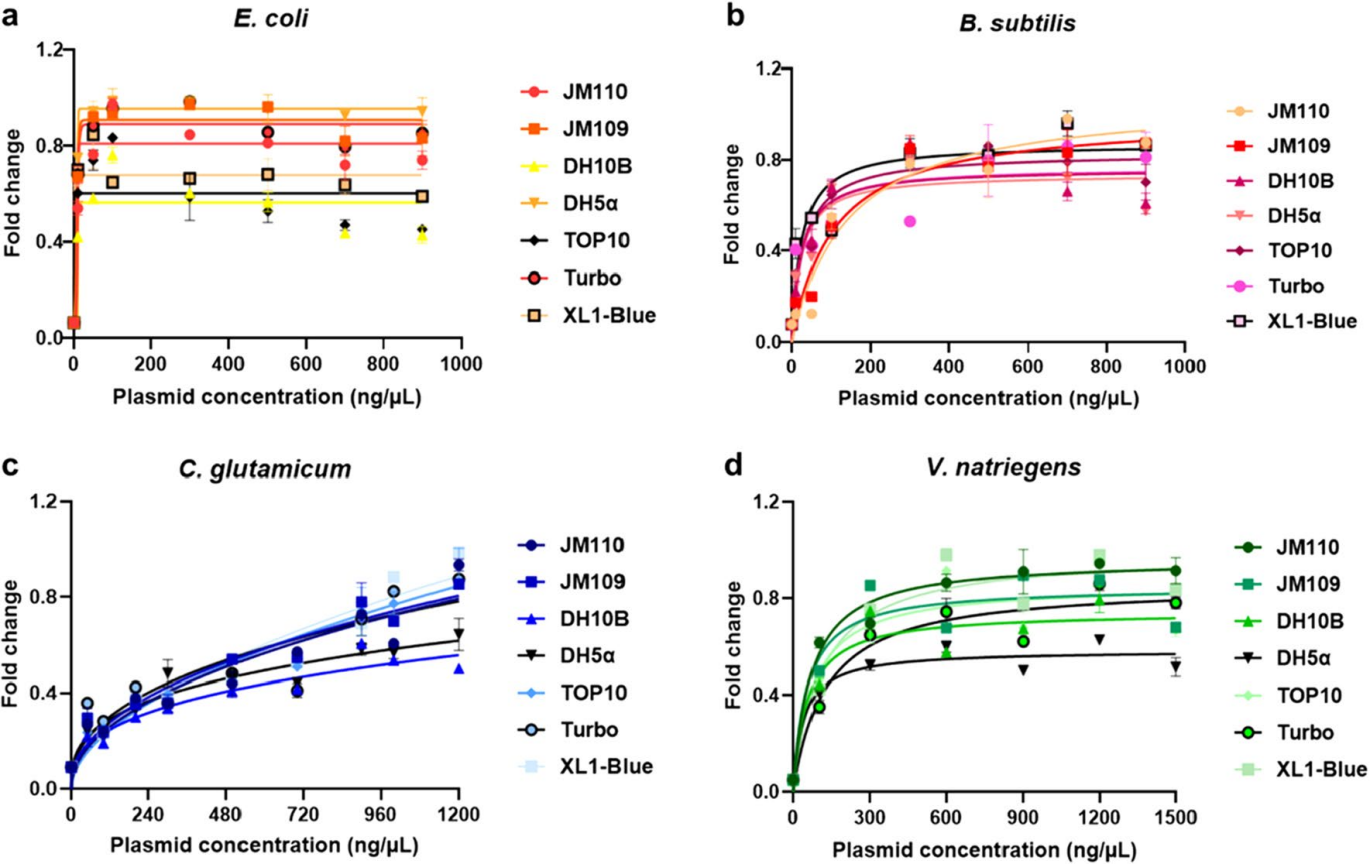
Screening of competent cells and DNA concentrations in *E. coli*, *B. subtilis*, *C. glutamate* and *V. natriegens* CFPS systems. **a** *E. coli* CFPS system. **b** *B. subtilis* CFPS system. **c** *C. glutamicum* CFPS system. **d** *V. natriegens* CFPS system. The data were normalized. The mean and standard deviations are shown (N = 3). The number "1" of the normalized treatment represented the optimal fluorescence value of each factor in each CFPS system

To explore the optimal conditions of target gene expression and obtain higher protein expression level, different plasmid sources were used to test their influences on protein expression. Plasmid transcription is influenced by host background and regulated by host factors. The difference in host background can affect the transcription mode of plasmid skeleton and auxiliary genes (Carroll and Wong 2018; Miyakoshi et al. 2009). The restrictive modification systems of the host can recognize and destroy exogenous DNA. For example, methylation of Dam^+^ and Dcm^+^ interferes with the cleavage of DNA cloned and propagated in *E. coli* strains, and also affects the efficiency of plasmid transformation (Russell and Zinder 1987). In addition, the host recombination system can catalyze the rearrangement of recombinant molecules, affecting the integrity of DNA (Weinstock et al. 2016). Certain genes in the host (e.g., Lon) encode specific proteases that cause degradation of the recombinant protein, reducing protein production (Phillips et al. 1984). As host cells might affect the transcription functions of plasmids, seven common *E. coli* competent cells (JM110, JM109, DH10B, DH5α, TOP10, Turbo, XL1-Blue) were selected for testing (Casali 2003). The data (Fig. 3) showed that the selection of competent cells had a significant effect on the protein yield of the system, with a nearly twofold difference in the yield between different competent cells. The optimal competent cell of *E. coli* system was DH5α, and the less effective competent cells were DH10B and TOP10 (Fig. 3a). The experimental data showed that for the *B. subtilis*, *C. glutamicum* and *V. natriegens* CFPS systems, the sfGFP expression reached a peak when XL1-Blue competent cell was used, and the use of DH5α competent cell resulted in low expression (Fig. 3bcd). These results indicated that DH5α was more suitable as a source of plasmids in *E. coli* system, which was conducive to protein expression. Moreover, the plasmids obtained from XL1-Blue were more suitable for the expression of *B. subtilis*, *C. glutamicum* and *V. natriegens* system proteins.

In addition, DNA concentration is the critical factor affecting the protein yield in CFPS reactions. Therefore, the next step was attempted to optimize protein expression by adjusting the plasmid concentration in cell-free reactions. It is hypothesized that an increase in plasmid concentration could lead to an increase in protein production. By increasing the plasmid concentration from 0 ng/μL to 1200 ng/μL (the range set by the pre-experiment), it was observed that there was a saturation point of plasmid concentration. The protein yield increased with the increase of plasmid concentration before reaching saturation point and tended to be stable after reaching the saturation point. From 100 ng/μL to the optimal plasmid concentration, protein yield could be increased by about 1–3 times (Fig. 3). Finally, the experimental data showed that for the *E. coli* CFPS system, the sfGFP expression reached a peak when 300 ng/μL plasmid DNA was provided as the template (Fig. 3a). The optimal plasmid concentration of *B. subtilis* system was 700 ng/μL (Fig. 3b). For the *C. glutamicum* CFPS system, the expression level of sfGFP was always on the rise in the selected plasmid concentration range, so the maximum value of the selected range was 1200 ng/μL (Fig. 3c). For the *V. natriegens* CFPS system, the sfGFP expression reached the peak when the 600 ng/μL plasmid DNA was provided as the template (Fig. 3d). However, the optimal plasmid concentrations required by *B. subtilis*, *C. glutamicum* and *V. natriegens* systems were all higher than those of *E. coli* systems, indicating that these three systems required more transcription elements to support the transcription and translation processes. The reason also could be that the transcription and the translation were inefficient, or the RNAs were easily degraded. In the future, to decrease the plasmid usage of these three systems, the genomes of the three model cells could be modified to enhance the transcription and translation process.

### The influence of RBS on different CFPS systems

It has been reported that there are significant differences in the initial translation efficiency and protein expression levels, with different intensities of RBS (ribosomal binding site) sequences (Shi et al. 2018). For this test, six RBS sequences were selected for each CFPS system to test the relationship between RBS and sfGFP yield. RBS sequences were designed according to RBS Library Calculator (Li et al. 2016; Salis 2011), and the six RBS sequences (Table 1) were equably selected from the minimum to the maximum translation initiation rates (see “Materials and methods” for detailed information). The fluorescence intensity of the system was quantified to screen RBS with different theoretical translation initiation rates, and finally, the influence of the designed RBS on the protein expression of the systems was obtained.

**Table 1:** RBS sequences designed by RBS Library Calculator

Bacterial specie	RBS number	RBS sequence	Translation initiation rate
*E. coli*	Original	GAAGGAG	6748.38
	1	CCCGAUGGGAUCACGCAUCUAAGG	0.13
	2	UCCGAUGCGACAAGGCAGGUCCUA	9.43
	3	UCCGAUGGGACAAGGCAGAUCCUA	38.37
	4	CCCGAUGCGAUCAAGGAUCUAAGG	158.33
	5	CCCGAUGGGAUCAAGAAUGUAAGG	642.63
	6	UCCGAUGGGACAAAGGAGGUUGUA	67,421.00
*B. subtilis*	Original	GAAGGAG	9491.88
	1	UUAUAGACAUUGGACAGUUCCUCGUUA	0.35
	2	UUAUAGACAUUGGGAAGUUCCUCCUUA	32.55
	3	UUAUAGACAUUGGAAAGUUACUCCUUA	130.15
	4	ACAUAGACAUUGGAAAGUUACUUCUA	464.48
	5	ACGAUGUAGUGAAGGGGUUACUUAUA	2053.54
	6	ACAAUGUAGUGAAGGGGGUACUUAUU	189,477.64
*C. glutamicum*	Original	GAAGGAG	8338.00
	1	UUCGGUUUACUAAACGCGCCCUUAU	0.12
	2	UGCGGUUUACUAAACGGACCCUUAU	8.12
	3	UGCGGUUUACUAAAAGCACCCAUAU	32.02
	4	UUCAGUUGGUAAAGGGAACCCAUAU	95.11
	5	GUCAGAUGGUAAAAAGGAGUUAAUCC	416.15
	6	UAAUAGUCACUUUAAGGAGGUUUAU	50,737.39
*V. natriegens*	Original	GAAGGAG	6748.83
	1	CCGUAUUUUUUCGACGCCGGUAUCCU	0.15
	2	CAGUCUUUUUUCAGCGCAGGUAAACA	12.18
	3	CCGUAUUUUUUCAAGGCCGGUAACAU	50.95
	4	CCGUAUUUUUUCAAUGGCGGUAAUAU	201.77
	5	CCGUAUUUUUUCUACGGAGGUAUUCU	1184.25
	6	CCGUAUUUUUUCAAGGGAGGUUAUAU	31,367.00

**Fig. 4:**
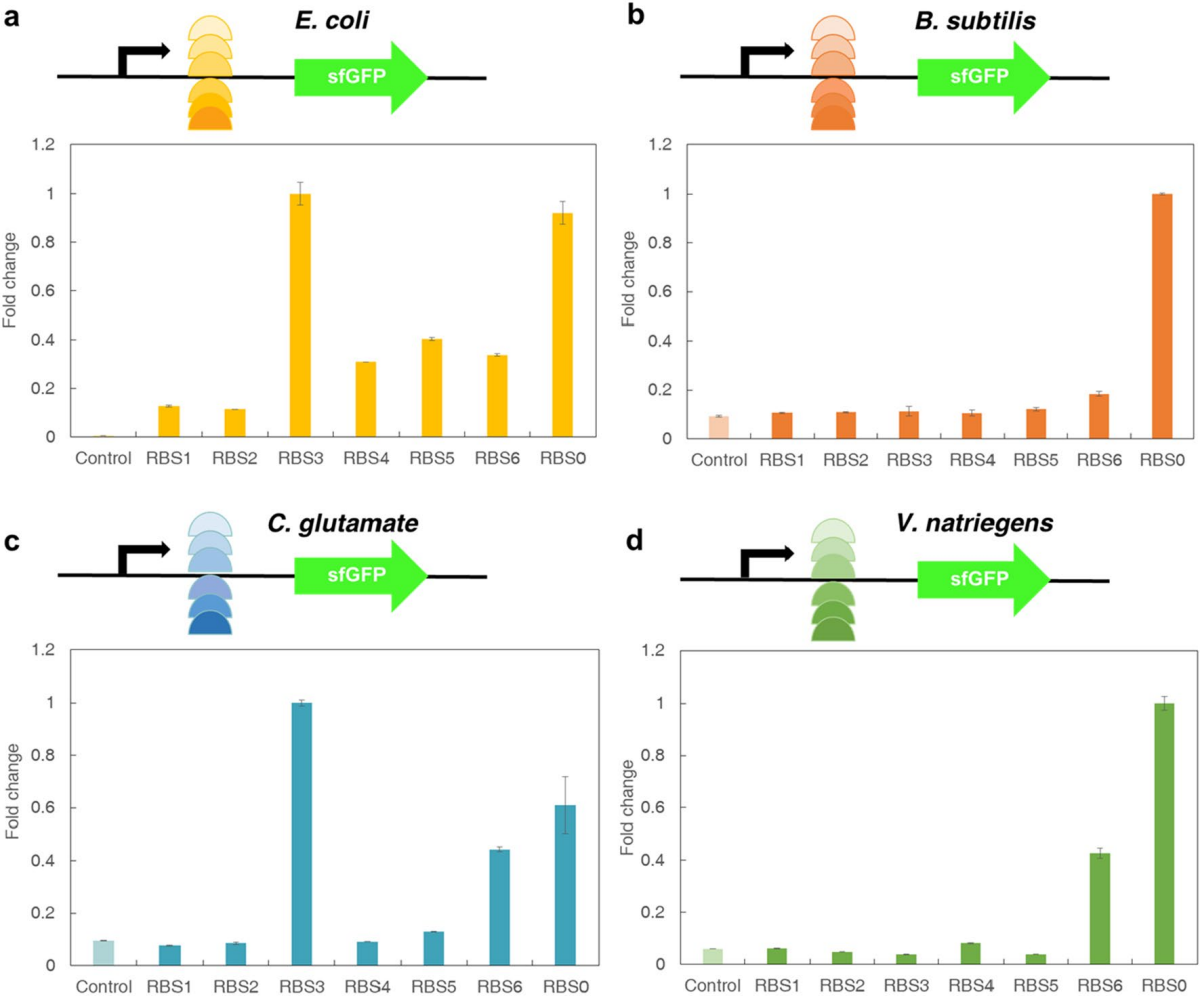
Effect of RBS on protein expression in *E. coli*, *B. subtilis*, *C. glutamate* and *V. natriegens* CFPS systems. **a** *E. coli* CFPS system. **b** *B. subtilis* CFPS system. **c** *C. glutamate* CFPS system. **d** *V. natriegens* CFPS system. The control is the background value of the systems, and the mean and standard deviations are shown (N = 3). The number "1" of the normalized treatment represented the optimal fluorescence value of each factor in each CFPS system

Theoretically, with the increase of the initial translation rate, the synthesis efficiency of sfGFP protein should be better, and the protein synthesis level of the system should be higher. However, the results were not regular, the protein synthesis level did not increase with the increase of the initial rate of RBS translation, and did not show a linear trend. As shown by the normalized experimental results, the new RBS designed for *E. coli* CFPS system (Fig. 4a) were all successfully expressed, but overall, most of the newly designed RBS did not have higher protein synthesis level than the original RBS, and only the protein synthesis level of RBS3 was slightly higher than the original RBS (RBS0). For the *B. subtilis* cell-free system (Fig. 4b), where none of the newly designed RBS sequences were successfully expressed. In addition, only RBS3 and RBS6 were successfully expressed in the *C. glutamicum* system (Fig. 4c), and the protein synthesis level of RBS3 was 40% higher than that of the original RBS0. For the *V. natriegens* system (Fig. 4d), none of RBS1-5 made protein expression. Although RBS6 made protein successfully expressed, it was still not as high as the protein synthesis level of the original RBS0. This indicated that there was a gap between the results predicted by the experimental theory and the test results. However, the theoretical prediction provided a certain reference for this experiment and helped to carry out the test.

Some of the designed RBS were not expressed probably due to the following reasons. First, the computer simulation itself had errors in predicting the RBSs. Second, the computer-designed RBS sequences for suitable hosts were more suitable for cells study. Third, the systems were not expressed well and did not present the expected results because the RBSs designed with a suitable host were not adapted to the promoter, vector or plasmid source. However, the results also showed that the CFPS system could provide a very fast method for screening and testing libraries. Therefore, the specific impact of RBSs on CFPS systems still needs further study.

### Optimization of cell-free system reagent components

**Fig. 5:**
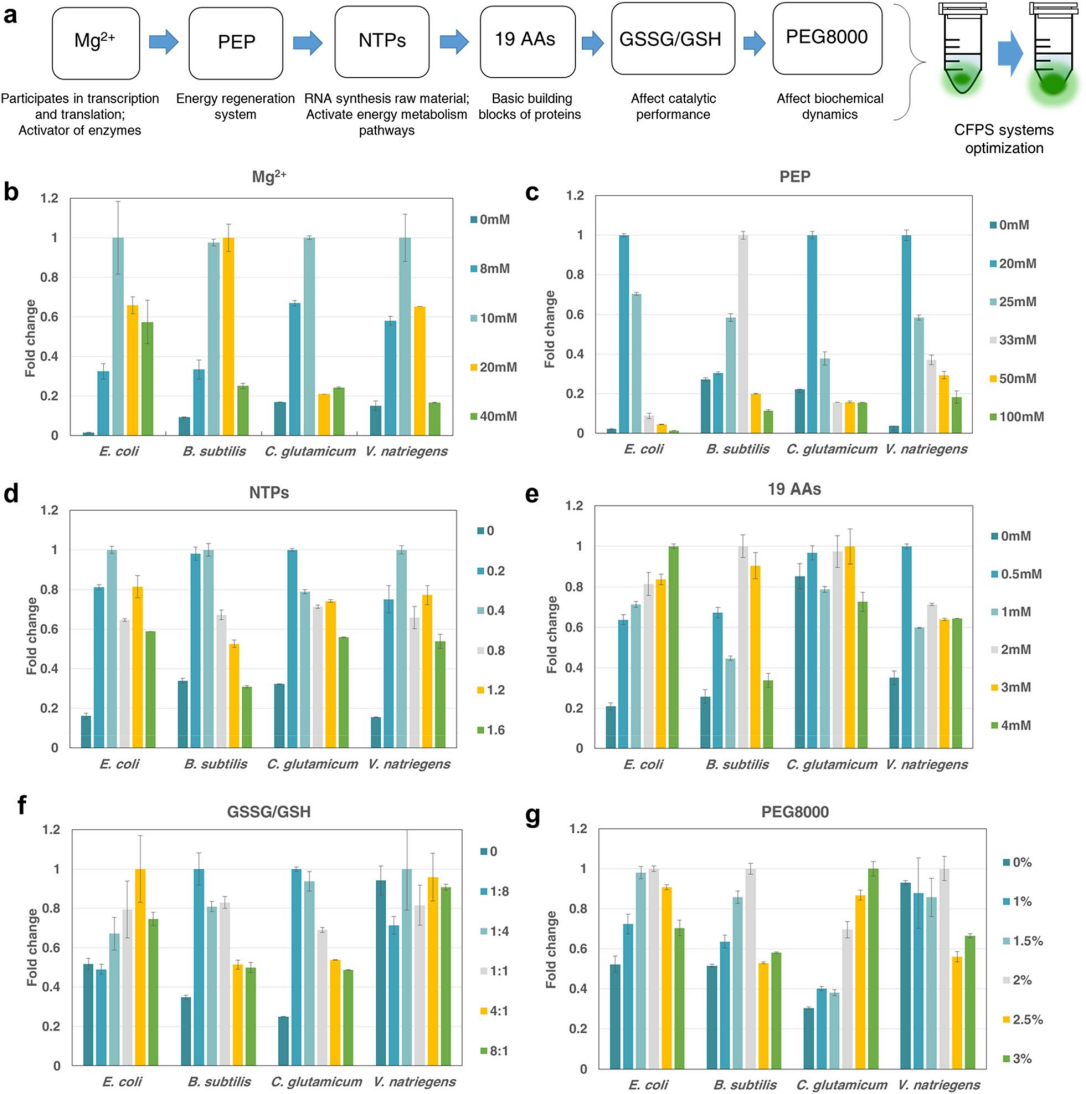
Effect of system reagent components on *E. coli*, *B. subtilis*, *C. glutamate* and *V. natriegens* CFPS systems. **a** Optimization process of reagent components in the system. **b** Mg^2+^. **c** PEP. **d** NTPs. **e** 19 AAs. **f** Redox environment. **g** PEG8000. Normalized treatment of sfGFP protein synthesis levelobtained by the system when using each reagent at a specified concentration. The mean and standard deviations are shown (N = 3). The number "1" of the normalized treatment represented the optimal fluorescence value of each factor in each CFPS system

The next study was to optimize reagent components of the CFPS system. The extract-based CFPS system utilizes some essential substrates (including amino acids, energy substrates, cofactors, and salts) that are essential for protein synthesis from CFPS systems (Carlson et al. 2012). All these components affect protein synthesis to a certain extent. *E. coli, B. subtilis, C. glutamate* and *V. natriegens* CFPS systems may have different requirements for small molecules and other reagents. Therefore, the effects of several important system parameters (Mg^2+^, PEP, NTPs, amino acids, oxidized reductant and PEG8000) on the yield of sfGFP in different CFPS systems were explored (Fig. 5a) (Des Soye et al. 2018). It is hypothesized that by modifying the *E. coli* CFPS system components of known protocol (Jiang et al. 2021) and conducting new experiments on different systems, the optimal system components suitable for different CFPS systems was obtained. A uniform gradient was designed (Additional file 1: Table S2) by varying the concentrations of several key components according to the known component combination. According to the gradient experiment, the optimal number of different components was selected. Meanwhile, the concentration of all other components in CFPS remained constant during the optimization process.

The effect of magnesium ions (Mg^2+^) on protein expression in different CFPS systems was first studied. Mg^2+^ can be used to balance the charges generated by nucleic acid phosphate groups and other anionic species in the system. It can also affect the interactions between proteins and nucleic acids in biological processes such as protein synthesis, and can act as an activator of some enzymes (RNA polymerase and carbamyl tRNA synthase) (Jiang, et al. 2021; Levine et al. 2019). It was found that the concentration of Mg^2+^ had a great influence on the expression of sfGFP in CFPS systems (Fig. 5b). The effect of 10 mM and 20 mM Mg^2+^ on protein expression was similar in *B. subtilis* system. However, the expression of 20 mM Mg^2+^ in the other three systems was much lower than that in 10 mM Mg^2+^. It could be because the transcription and translation process of these three systems was more sensitive to the demand of Mg^2+^. The transcription and translation process of the *B. subtilis* system also kept working efficiently at 20 mM Mg^2+^, but higher concentrations of Mg^2+^ led to a decrease in protein expression. *E. coli*, *B. subtilis*, *C. glutamate* and *V. natriegens* CFPS systems had the highest protein activity when the final concentration of magnesium glutamate was 20 mM, 10 mM, 10 mM and 10 mM, respectively.

In addition, providing sufficient energy for the transcription and translation processes in a CFPS system is also essential for efficient protein synthesis. Phosphoenolpyruvate (PEP) can be used as an energy regeneration system to activate the translation response in the CFPS system (Failmezger et al. 2018). It had a great influence on the protein expression of *E. coli* and *V. natriegens* CFPS system (Fig. 5c), and the maximum protein synthesis level differed by dozens of times. High concentration of PEP (33 mM) was more suitable for the protein expression of *B. subtilis*. Lower concentration of PEP (20 mM) was more suitable for *E. coli, C. glutamate* and *V. natriegens* CFPS systems.

NTPs Mix is a hybrid system, including ATP, CTP, GTP, UTP, CoA, putrescine, spermine, NAD, tRNA and folic acid. NTPs are added to provide raw material for transcription. The addition of purified tRNA stimulates the translation process, and the addition of NAD^+^ and CoA activates the pathway from pyruvate to acetylphosphate to stimulate energy metabolism. In this study, with the increase of NTPs content, the protein expression of the four systems showed a trend of increasing first, and too much NTPs was more detrimental to the protein expression of systems (Fig. 5d). The reason why excessive PEP and NTPs decreased protein expression in the systems might be that excessive phosphate was produced, which led to phosphate accumulation and was detrimental to protein synthesis. Compared with the three CFPS systems, it was found that the protein expression process of the *B. subtilis* CFPS system needed to provide more energy substances to support. *E. coli*, *B. subtilis*, *C. glutamate* and *V. natriegens* CFPS systems exhibited the highest protein expression when 0.4 μL, 0.4 μL, 0.2 μL and 0.4 μL NTPs were added to each reaction (20 μL), respectively.

Amino acid is an essential substrate and key component of protein synthesis. In addition, certain amino acids are active participants in central metabolic pathways. According to the experimental results (Fig. 5e), for the *C. glutamicum* CFPS system, different amino acid concentrations had no significant effect on protein synthesis. The protein expression of *C. glutamicum* CFPS system was relatively high when the amino acid concentration was 3 mM. However, in *E. coli*, *B. subtilis* and *V. natriegens* CFPS systems, different concentrations of amino acids resulted in significant difference in the protein expression levels of the systems. Among them, the *E. coli* system required more amino acids to support the translation process. The protein expression levels of *E. coli*, *B. subtilis* and *V. natriegens* systems reached the peak at 4 mM, 2 mM and 0.5 mM amino acids, respectively.

Next, the redox environment in the optimized reaction mixture was studied (Fig. 5f). The oxidation environment in the system is also a key factor affecting protein synthesis, because it can affect the catalytic performance of the system and also affect the formation of disulfide bonds in some proteins (Yin and Swartz 2004). The redox environment was regulated by the oxidized and reduced glutathione (GSSG and GSH). It was found that the addition of oxidizing reducing agent or the redox degree of the environment had little effect on the protein expression of the *V. natriegens* CFPS system. In addition, the oxidation environment was more conducive to protein synthesis for *E. coli* system, while the reductive environment was more preferred for *B. subtilis* and *C. glutamicum* systems.

Finally, the tested influence factor was molecular crowding degree. Molecular crowding, as a natural state of cells, affects the rate of diffusion and binding of molecules in the environment, which in turn affects protein synthesis (Tan et al. 2013). To restore the state of molecular crowding, a crowding agent, PEG8000 could be added to the CFPS system to simulate the degree of intracellular molecular crowding. It could be seen from the experimental data (Fig. 5g) that the molecular density in different cell-free environments still had a certain influence on protein expression. Among them, the addition of PEG8000 had little effect on the *V. natriegens* system, indicating that the molecular crowding degree of the *V. natriegens* system itself was also suitable for protein synthesis. The *C. glutamicum* system may be due to the low density of CFPS system compared with other systems, which required the addition of high concentration of PEG800 to maintain the crowding degree of the system, thus improving the macromolecular binding efficiency of the system. In addition, the high molecular density was not conducive to protein expression in *E. coli*, *B. subtilis* and *V. natriegens* systems. This might be due to the increased viscosity of the crowding agent, the high molecular density in the system, which increased the burden of the system and led to the decrease of protein expression efficiency. The protein synthesis efficiency of *E. coli*, *B. subtilis* and *V. natriegens* systems reached the highest, when 2% PEG8000 was added, and the optimal PEG8000 concentration of *C. glutamicum* system was 3%.

The correlation analysis results of different reagent components of four CFPS systems (Additional file 1: Table S5) showed that the influence degree of the six reagent components on the protein expression of four CFPS systems from large to small was as followed. *V. natriegens*: PEG8000, Mg^2+^, PEP, GSSG/GSH, NTPS, 19 AA; *B. subtilis*: NTPS, PEP, 19 AA, GSSG/GSH, Mg^2+^, PEG8000; *C. glutamicum*: PEG8000, PEP, Mg^2+^, 19 AA, GSSG/GSH, NTPS; *E. coli*: 19 AA, GSSG/GSH, PEG8000, PEP, Mg^2+^, NTPS. By optimizing and comparing the influencing factors of different CFPS systems, the protein synthesis level of the system can be improved, and some factors with greater influence can be selected for targeted optimization, so as to eliminate the ineffective factors and improve the optimization efficiency.

The CFPS systems can quickly and conveniently add different concentrations of reagents to screen according to the needs of systems based on different hosts. This ability to adapt to the rapid optimization of each system is the outstanding advantage of the cell-free system. The protein yield of the system can be improved by directly optimizing the corresponding influencing factors. After a series of optimization work, the best components of the four CFPS systems were determined, as shown in Additional file 1: Table S3. The experimental results (Table 2) showed that the total protein synthesis of the sfGFP was improved in all four CFPS systems after optimization. The total sfGFP protein yield of the optimized *E. coli*, *B. subtilis*, *C. glutamate* and *V. natriegens* systems could reach 1435.4 mg/L, 560.9 mg/L, 384.6 mg/L, and 725.3 mg/L, respectively. By comparing the four optimized CFPS systems, it could be found that the expression levels of the constructed *B. subtilis*, *C. glutamate* and *V. natriegens* CFPS systems were still lower than that of the *E. coli* CFPS system, indicating that these three CFPS systems still need to be improved step by step.

**Table 2:** Final protein expression levels of the four CFPS systems

Bacterial species	Unoptimized system total protein (mg/L)	Optimized system total protein (mg/L)	Key factors of protein expression
*V. natriegens*	569.7 ± 4.1	725.3 ± 22.6	Plasmid source, plasmid concentration, PEG8000, Mg^2+^, PEP
*C. glutamicum*	287.2 ± 25.5	384.6 ± 7	Plasmid source, plasmid concentration, codon, NTPs, PEP, 19 AA
*B. subtilis*	306.1 ± 9.6	560.9 ± 8.7	Plasmid source, plasmid concentration, PEG8000, PEP, Mg^2+^
*E. coli*	936.1 ± 8.4	1435.4 ± 37	Plasmid source, plasmid concentration, codon, 19 AA, GSSG/GSH, PEG8000

### Expression of SARS-CoV-2 RBD protein and the effect of surfactants on protein solubility

**Fig. 6:**
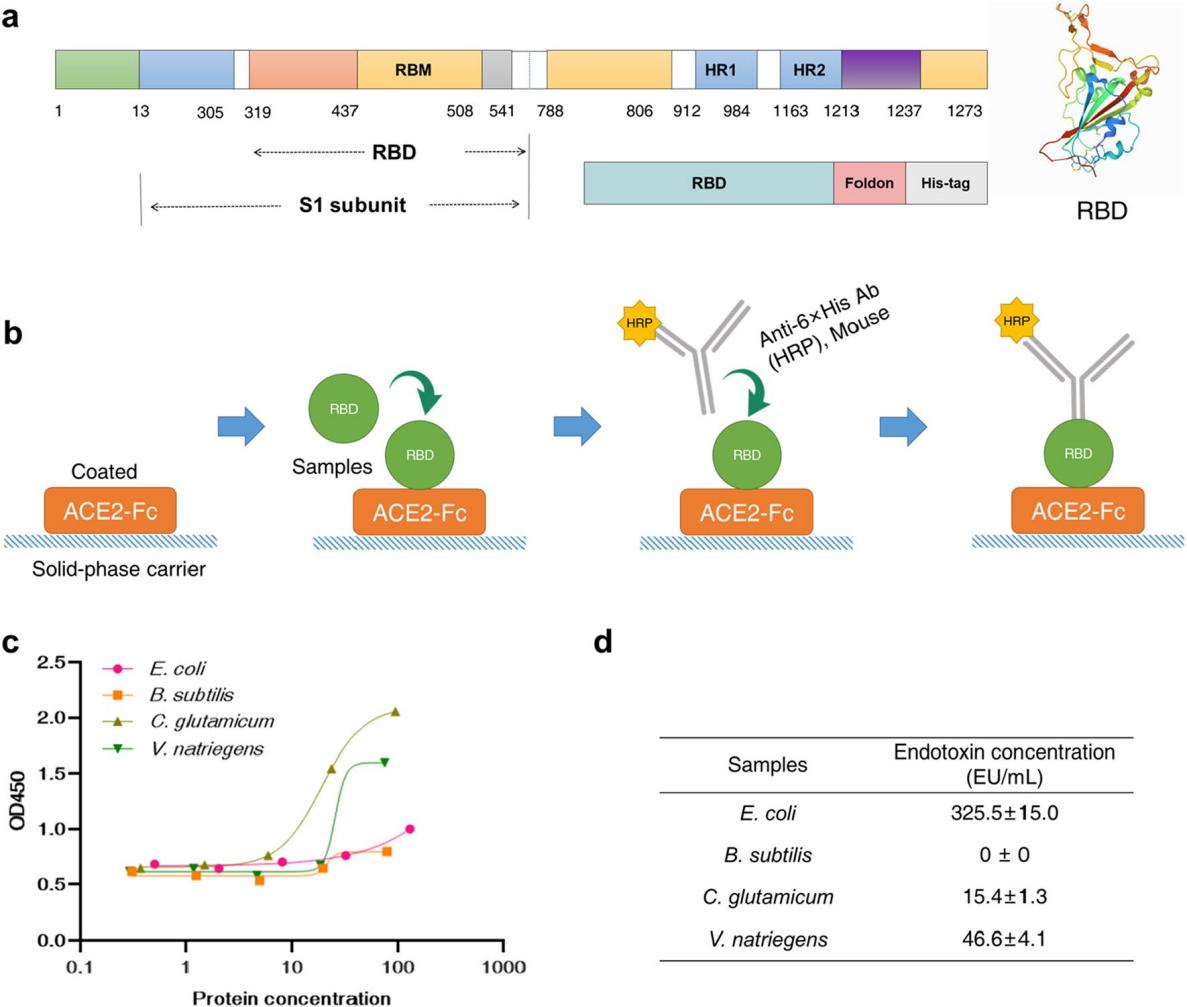
The *E. coli*, *B. subtilis*, *C. glutamicum* and *V. natriegens* CFPS systems as a platform for pharmaceutical protein synthesis. **a** Protein structure diagrams and sequence design diagrams of RBD protein (PDB: 6VW1). **b** Schematic diagram of ELISA principle. **c** Absorbance of four system protein samples with different concentrations at 450 nm. **d** Endotoxin detection of extracts in the four CFPS systems

To better demonstrate that the CFPS systems could be a platform for therapeutic protein expression, the four optimized CFPS systems were used to express the RBD protein of SARS-CoV-2. SARS-CoV-2 is the most concerned virus at the moment because of its powerful infectivity and lethality (Wang et al. 2020; Zhai et al. 2020). S protein plays the most important role in the process of virus attachment, fusion and entry into host cells, and it acts on antibodies and is a target for the development of inhibitors and vaccines. The S protein of SARS-CoV-2 contains a receptor-binding domain (RBD) that specifically recognizes the receptor; thus RBD is a key target for antiviral compounds and antibodies (Fig. 6a) (Shang et al. 2020). Studies on the synthesis of RBD protein may contribute to the development of SARS-CoV-2 therapy. Therefore, the RBD-Foldon protein was selected as the target protein to be expressed in this study.

First, the expression of RBD-Foldon was proceeded to be tested by using the *V. natriegens* CFPS system. The results showed that RBD-Foldon could be successfully expressed, but the solubility of the protein was very low (Additional file 1: Fig. S1a). To improve protein solubility, two nonionic surfactants (Tween 20 and Brij-35) were used to test their effects on protein solubility in the CFPS systems. The nonionic surfactant interacts with the hydrophobic portion of the protein, thereby exposing hydrophilic groups present in both molecules (Aguirre-Ramírez et al. 2021), which results in an increase in the hydrophilicity of the nonionic surfactant–protein complex, thereby reducing protein aggregation (Additional file 1: Fig. S2). The results were verified by protein western blotting, which showed that the addition of Tween 20 did not have a significant effect on protein solubility (Additional file 1: Fig. S1c). However, after the addition of Brij-35, the protein solubility increased significantly. Therefore, Brij-35 surfactant could effectively increase the solubility of protein (Additional file 1: Fig. S1b). Then, the RBD-Foldon was expressed in *B. subtilis*, *C. glutamicum* and *E. coli* CFPS systems (Additional file 1: Fig. S3). The three CFPS systems also added Brij-35 surfactant to test the protein solubility because of the better results of the *V. natriegens* system tested. It was clear that the RBD-Foldon protein was successfully expressed in *B. subtilis*, *C. glutamicum* and *E. coli* CFPS systems, and the Brij-35 surfactant could significantly increase the protein solubility of the three CFPS systems. Finally, RBD-Foldon protein was expressed and quantified in four CFPS systems supplemented with Brij-35 (Additional file 1: Fig. S4).

To verify the functional activity of the proteins, the RBD-Foldon protein produced by the four CFPS systems were verified by ELISA assay (Fig. 6b). The Anti-His_6_-Mouse Ab-HRP was titrated on the pre-coated, pre-blocked ACE2-Fc ELISA plate to determine the optimal dilution of the proteins to be used in the validation of the ELISA. From Additional file 1: Fig. S5, it could be seen that the color changed significantly after color development, which proved that the RBD-Foldon proteins produced by the four systems could bind to ACE2, and the binding rate of the samples was the highest at the original concentration. As can be seen from Fig. 5c, compared with the protein contents produced by the four CFPS systems (Additional file 1: Table S4), although the protein contents produced by *V. natriegens* and *C. glutamicum* CFPS systems were less than that of *E. coli*, these two CFPS systems had stronger protein production activity. Therefore, the RBD-Foldon proteins produced by *E. coli, B. subtilis, C. glutamicum and V. natriegens* CFPS systems could be verified by ELISA and were functionally active.

Next, the endotoxin of cell extracts from all four systems was tested. The results (Fig. 6d) showed that in the same volume of the four cell extracts, the endotoxin of *B. subtilis*, *C. glutamicum*, *V. natriegens* cell extract was significantly lower than that of *E. coli*. As we known, *B. subtilis* and *C. glutamicum* are Gram-positive bacteria and do not produce endotoxin. Part of the error might be due to the accumulation of lipopolysaccharide caused by microorganisms in the environment during the preparation process, which led to higher detection results. Therefore, the *V. natriegens*, *C. glutamicum*, *B. subtilis* CFPS systems were safer for the production of therapeutic proteins. Compared with the *E. coli* CFPS system, the *B. subtilis, C. glutamicum and V. natriegens* CFPS systems had more potential for the design and production of pharmaceutical proteins in the future.

## Conclusion

*V. natriegens*, *B. subtilis*, *C. glutamicum* and *E. coli* are four well established model organisms with high potential and industrial value. In this study, *E. coli*, *B. subtilis*, *C. glutamicum* and *V. natriegens* CFPS systems were constructed, and their related process parameters were optimized. Through the comparison and analysis of the four CFPS systems, the advantages and important influencing factors of different systems were obtained. For example, *B. subtilis* and *V. natriegens* did not require codon optimization. The transcriptional and translation efficiency of *V. natriegens*, *B. subtilis* and *C. glutamicum* system were lower than that of *E. coli*. The endotoxin levels in the cell extracts of *B. subtilis*, *C. glutamate* and *V. natriegens* systems were much lower than that of *E. coli*. They not only expanded the potential options for in vitro protein production, but also increased the application range of the systems.

According to the characteristics of different systems, a suitable system could be selected for applications. For example, all four CFPS systems were suitable for high-throughput screening of proteins. The endotoxin levels in the cell extracts of *B. subtilis*, *C. glutamate* and *V. natriegens* systems were much lower than that of *E. coli*, which could reduce the burden of downstream processing in the biopharmaceutical industry. Therefore, these three CFPS systems have greater potentials for the design and production of future drug proteins. However, due to the relatively low levels of protein synthesis in *B. subtilis*, *C. glutamate* and *V. natriegens* systems compared to *E. coli* system, some applications that require a higher protein yield, such as cell-free biosensors that require a stronger output signal, are still suitable for *E. coli* system.

The expression levels of *B. subtilis*, *C. glutamate* and *V. natriegens* CFPS systems were still lower than that of *E. coli* CFPS system, which might be due to inappropriate plasmid sources. Jurek Failmezger et al*.* (Failmezger, et al. 2017) found that plasmids with the same cellular background as the CFPS system were well expressed. In this study, *E. coli*-competent cells were used to extract plasmids, and pET-23a vector was used to synthesize the proteins, which might have certain limitations on the synthesis of different CFPS systems. Therefore, plasmids can be extracted from *V. natriegens*, *C. glutamate* and *B. subtilis*, respectively, and various expression vectors can be designed to further increase the expression of CFPS systems. In addition, studies on the metabolic pathways, gene circuits and diagnostic tools (Cho and Lu 2020; Lin et al. 2021; Lin et al. 2020a, b; Yang and Lu 2020) in the *E. coli* CFPS system can be tried in the other three systems, and a diversified CFPS platform can be developed further to expand the application scope of the CFPS systems.

## Materials and methods

### Bacterial strains and plasmids

The strains used in this study were *Escherichia coli* BL21 STAR(DE3), *Bacillus subtilis* 168, *Corynebacterium glutamate* MB001(DE3) and *Vibrio natriegens* Vmax (Additional file 1: Table S1). The sfGFP-6 × His plasmid, codon optimization plasmids and pET24a( +)-RBD-His plasmid were synthesized in GENEWIZ Company. All the plasmids used in this experiment were sequentially verified. More detailed information is shown in Additional file 1: Table S6.

### RBS library design

Different RBS sequences were designed for each CFPS system to replace the original RBS sequence between the T7 promoter sequence and the initiation of sfGFP gene. RBS sequences were designed according to different hosts by the RBS library calculator (De Novo DNA: RBS Library Calculator) (Reis and Salis 2020; Salis et al. 2009). Because the range of translation initiation rates were very wide, so six RBS sequences (Table 1) were equably selected from the minimum to the maximum translation initiation rates. The designed RBS replaced the original RBS by site-specific mutagenesis. Then, the sequences were sent to the sequencing company to verify.

### RBD-Foldon protein design

Foldon was added to the C-terminal end of the RBD designed in this study. The addition of Foldon allowed the formation of trimers after RBD expression, thus enhancing immunogenicity (Vogel et al. 2021).

### Cell culture

To prepare the cell-free extracts (Gao et al. 2019; Jiang, et al. 2021), the cells of *Bacillus subtilis* 168, *Corynebacterium glutamate* MB001(DE3) and *Vibrio natriegens* Vmax were resurrected from glycerin to the antibiotic-free LB plate. After inoculation, the colonies were inoculated in 5 mL LB medium (first grade seed solution) and incubated overnight at 30 °C (200 rpm). *Escherichia coli* BL21 star (DE3) was cultured in ampicillin medium and at 37 ℃. The culture was diluted (1:20), added into a bottle containing 200 mL 2 × YTP medium (secondary seed solution), and shaken at 30 ℃/37 ℃ (200 rpm) to OD at 0.8 ~ 1. Then the culture was diluted (1:20) and added into a bottle containing 2 L 2 × YTP medium (tertiary seed solution). After shaking at 30 ℃/37 ℃ (200 rpm) until the end of the logarithmic growth period, the cells were harvested. By comparing the growth rate of the four bacteria, *E.coli* and *V. natriegens* could reach the collecting stage faster. The bacterial solution was centrifuged at 8000 rpm, at 4 ℃, for 30 min. The *Bacillus subtilis* produced spores that made it difficult to centrifuge, so that the centrifugation speed could be slightly increased. For the same volume of culture, there were much fewer bacteria in *B. subtilis* and *C. glutamate* than that in *E. coli* and *V. natriegens*. The bacteria were resuspended with crushing buffer S30A (14 mM Mg-glutamate, 60 mM K-glutamate, 50 mM Tris, pH 7.7) and transferred into a 50-mL pre-weighing Falcon tube. The suspension was centrifuged at 8000 rpm at 4 ℃ for 10 min.

### Extract preparation

Added 1 mL of S30A buffer for each gram of bacteria body, suspended again, and waited for use. An appropriate amount of ice water mixture was added into the high-pressure crushing apparatus to create a low-temperature environment and prevent overheating in the crushing process, which could affect the activity of cell extracts. Bacteria were crushed 2–3 times with a high-pressure crushing apparatus (15,000 Pa). The broken samples were centrifuged at 10,000 rpm at 4 ℃, 30 min. After centrifugation, the supernatant was transferred to the new BD tube, and 3 µl of 1 M DTT were added to each 1 mL solution. Then wrapped in foil, placed in a shaker at 37 ℃ away from light, and incubated at 120 rpm, 80 min. After incubation, the samples were centrifuged at 10,000 rpm, 4 ℃, 30 min, and the supernatant was retained. The supernatant was added to the dialysis bag of MWCO 6–8 kDa, and the dialysis buffer S30B (14 mM Mg-glutamate, 60 mM K-glutamate, 5 mM Tris, pH 8.2) was used for dialysis at 4 ℃, 4 h. The liquid in the dialysis bag was transferred to the BD tube, centrifuged at 10,000 rpm, 4 ℃, 30 min, and the supernatant was retained. The supernatant was separated on ice, and stored in the refrigerator at − 80 ℃ after rapid freezing of liquid nitrogen until used.

### Standard cell-free protein synthesis reaction

The standard CFPS reaction was conducted in a 1.5 mL EP (Eppendorf) tube at a volume of 20 µL, incubated overnight (about 13 h) at 30 °C. Then the protein synthesis level was detected with a microplate analyzer. The standard reaction mixture consists of the following components (Gao et al. 2019; Jiang et al. 2021): 300 ng/mL plasmid DNA, 175 mM potassium glutamate, 10 mM ammonium glutamate, 2.7 mM potassium oxalate, 100 mM magnesium glutamate, 2.5% PEG8000, 1 M PEP solution, 50 mM 19 amino acids, 25 × NTP Mix (1 mM puttamine, 1.5 mM spermidine, 0.33 mM NAD, 1.2 mM ATP, 0.86 mM CTP, 0.86 mM GTP, 0.86 mM UTP, 0.27 mM CoA, 170 g/mL tRNA) and ddH_2_O.

### ELISA

Each ELISA plate was covered with 2 μg/mL antigen (diluted with coated solution) 50 μL, and incubated overnight at 4 ℃ (sealed film). The next day, each well was washed with 200 μL PBS 3 times. Each well was sealed with blocking solution 200 μL at room temperature for 1 h. After sealing, each well was washed with 200 μL PBS 3 times. The sample was diluted with blocking solution (gradient dilution), 50 μL was added to each well (do not add to the wall), and incubated at room temperature for 3 h (seal the film). Then washed with 200 μL PBS 3 times per well. Added 0.2 μg/mL His Ab-HRP antibody 50 μL to each well (diluted with blocking solution), and incubated at room temperature for 1 h (sealed film). Washed with 200 μL PBS 3 times per well. Added 100 μL TMB solution to each well, waited for color change (the color turned blue if there is antigen–antibody binding). After the color remained the same, 100 μL stop solution (2 M sulfuric acid) was added to each well to terminate the solution, and then read at 450 nm with a microplate meter. First, the commercial standard RBD protein was tested by ELISA, which proved that RBD protein could be successfully bound to ACE2 protein. Then, the RBD proteins produced by the four CFPS systems were validated by ELISA.

### Gene expression analysis

Fluorescence output representing gene expression level was measured online in an infinite M200PRO (TECAN) by fluorescence (excitation filter 485 nm, emission 525 nm). When the cell-free reaction was completed, 10 µL sample and 190 μL water was mixed and read in a black, flat-bottomed 96-well analysis plate (CORNING). Before each reading cycle, the plate was shaken.

### Endotoxin analysis

The endotoxin of extract samples was detected using the bacterial endotoxin (ET) ELISA Kit (Shanghai mlbio Co.) and quantified according to the standard curve (Additional file 1: Fig. S6).

### Protein quantification

Western blot analysis was performed on the sample protein and standard gradient protein. The standard curve established by quantitative standard sfGFP protein sample was used, and the gray value was converted to protein concentration calculated by ImageJ software.

## Supplementary Information

**Additional file 1: Fig. S1.** Western blot analysis of the RBD-Foldon in the *V. natriegens* CFPS system. a. RBD-Foldon expression of the original *V. natriegens* system; b. RBD-Foldon expression of *V. natriegens* system after add the surfactant Brij-35; c. RBD-Foldon expression of the *V. natriegens* system after add the surfactant Tween 20. **Fig. S2.** Schematic diagram of the effect of surfactants on protein structure. **Fig. S3.** Western blot analysis of RBD-Foldon in the *C. glutamicum*, *B. subtilis* and *E. coli* CFPS systems. **Fig. S4.** Western blot and quantitative analysis of RBD-Foldon in *E. coli*, *B. subtilis*, *C. glutamicum* and *V. natriegens* CFPS systems. **Fig. S5.** Color change of four CFPS samples after TMB development and after termination of color development. **Fig. S6.** The standard curve of endotoxin. **Fig. S7.** The map of pET23a-sfGFP-6 × His. **Fig. S8.** The map of pET24a( +)-RBD-His. **Fig. S8.** Flow chart of experimental design. **Table S1.** The strains in this study. **Table S2.** The concentrations of components in original reference CFPS systems. **Table S3.** Addition gradient of CFPS system components. **Table S4.** Optimum conditions in the *V. natriegens*, *C. glutamicum*, *B. subtilis* and *E. coli* CFPS expression systems. **Table S5.** The significance analysis of different reagent components in four CFPS system.

## Data Availability

All data generated or analyzed during this study are included in this published article.

## Abbreviations

CFPS: Cell-free protein synthesis
RBS: Ribosomal binding site
sfGFP: Superfolder green fluorescent protein
PEP: Phosphoenolpyruvate
RBD: Receptor-binding domain
ELISA: Enzyme-linked immunosorbent assay
