# Supplementary material for: Development and comparison of cell-free protein synthesis systems derived from typical bacterial chassis
Source: Bioresour Bioprocess. 2021 Jul 6;8(1):58. doi: 10.1186/s40643-021-00413-2 (PMC8258279; doi:10.1186/s40643-021-00413-2)
Supplement: Supplementary file 1 — Additional file 1: Fig. S1. Western blot analysis of the RBD-Foldon in the V. natriegens CFPS system. a. RBD-Foldon expression of the original V. natriegens system; b. RBD-Foldon expression of V. natriegens system after add the surfactant Brij-35; c. RBD-Foldon expression of the V. natriegens system after add the surfactant Tween 20. Fig. S2. Schematic diagram of the effect of surfactants on protein structure. Fig. S3. Western blot analysis of RBD-Foldon in the C. glutamicum, B. subtilis and E. coli CFPS systems. Fig. S4. Western blot and quantitative analysis of RBD-Foldon in E. coli, B. subtilis, C. glutamicum and V. natriegens CFPS systems. Fig. S5. Color change of four CFPS samples after TMB development and after termination of color development. Fig. S6. The standard curve of endotoxin. Fig. S7. The map of pET23a-sfGFP-6 × His. Fig. S8. The map of pET24a( +)-RBD-His. Fig. S8. Flow chart of experimental design. Table S1. The strains in this study. Table S2. The concentrations of components in original reference CFPS systems. Table S3. Addition gradient of CFPS system components. Table S4. Optimum conditions in the V. natriegens, C. glutamicum, B. subtilis and E. coli CFPS expression systems. Table S5. The significance analysis of different reagent components in four CFPS system. [file 40643_2021_413_MOESM1_ESM.docx]

**Supporting Information**

**Development and comparison of cell-free protein synthesis systems derived from typical bacterial chassis**

**Liyuan Zhang^1,2,#^, Xiaomei Lin^2,#^, Ting Wang^2,#^, Wei Guo^1,*^, Yuan Lu^2,*^**

^1^ Department of Ecology, Shenyang Agricultural University, Shenyang 110866, Liaoning Province, China.

^2^ Key Laboratory of Industrial Biocatalysis, Ministry of Education, Department of Chemical Engineering, Tsinghua University, Beijing 100084, China.

^#^ These authors contributed equally to this work.

*** Corresponding authors:** 1999500011@syau.edu.cn (Wei Guo), yuanlu@tsinghua.edu.cn (Yuan Lu)


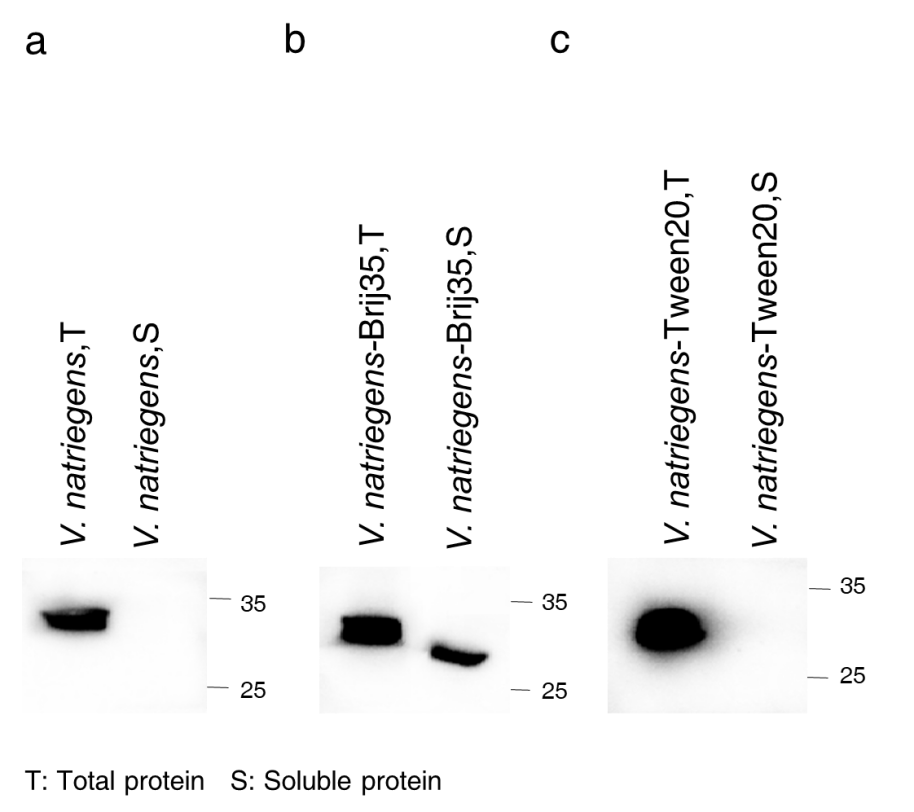


**Fig. S1.** Western blotting analysis of the RBD-Foldon in the *V. natriegens* CFPS system. a. RBD-Foldon expression in the original *V. natriegens* system; b. RBD-Foldon expression in *V. natriegens* system after adding the surfactant Brij-35; c. RBD-Foldon expression in the *V. natriegens* system after adding the surfactant Tween 20.


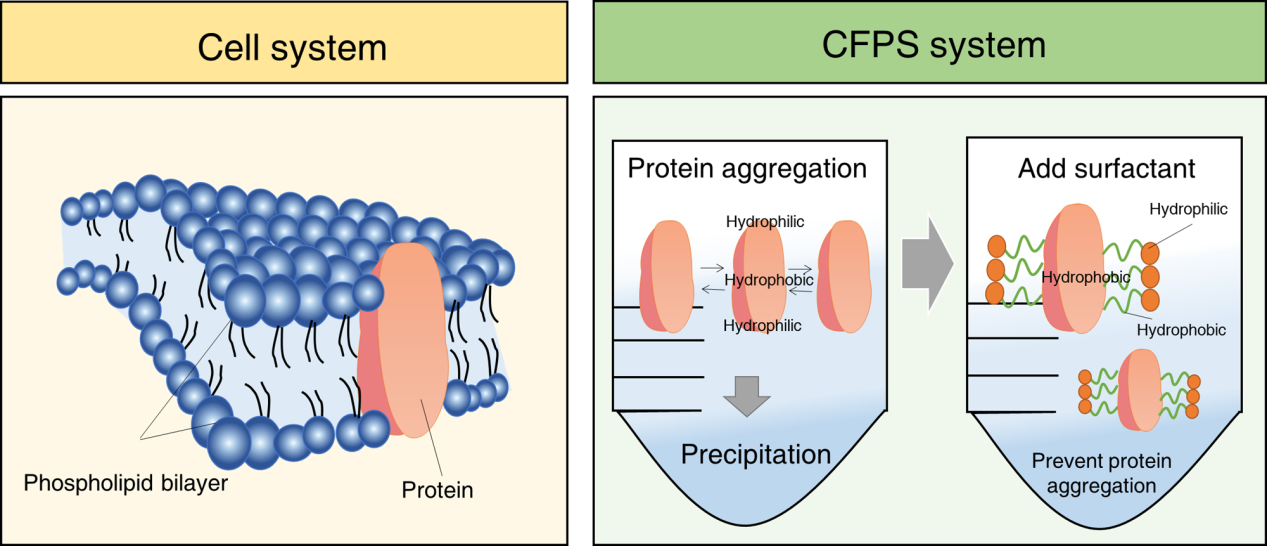


**Fig. S2.** Schematic diagram of the effect of surfactants on protein structure.


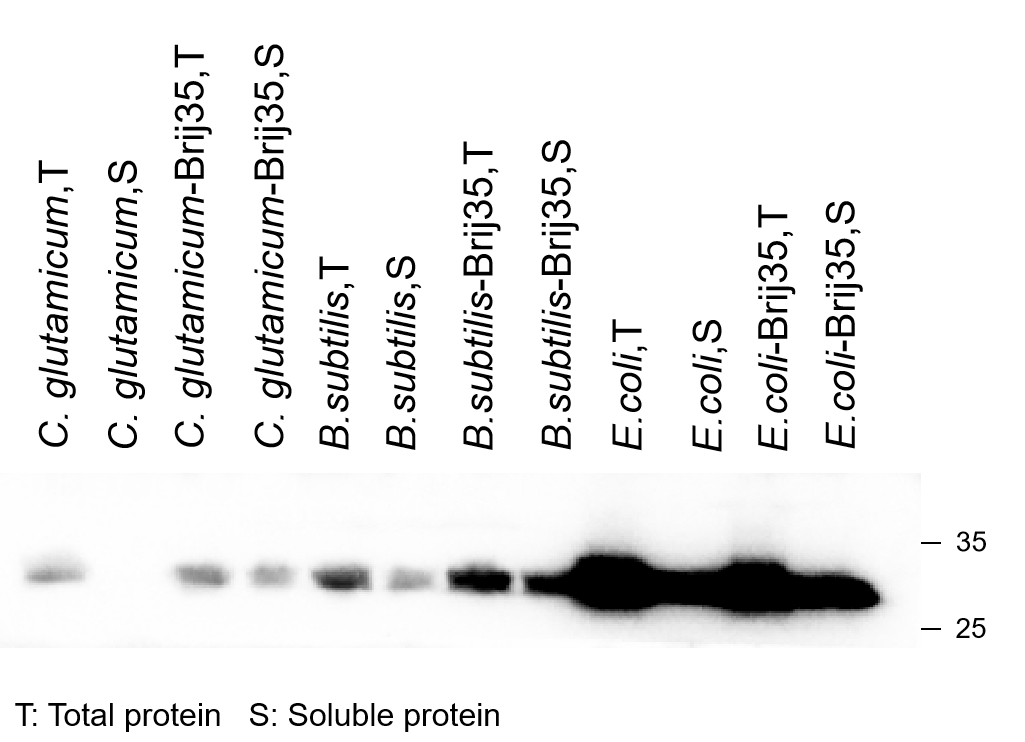


**Fig. S3.** Western blotting analysis of RBD-Foldon in the *C. glutamicum*, *B. subtilis* and *E. coli* CFPS systems.


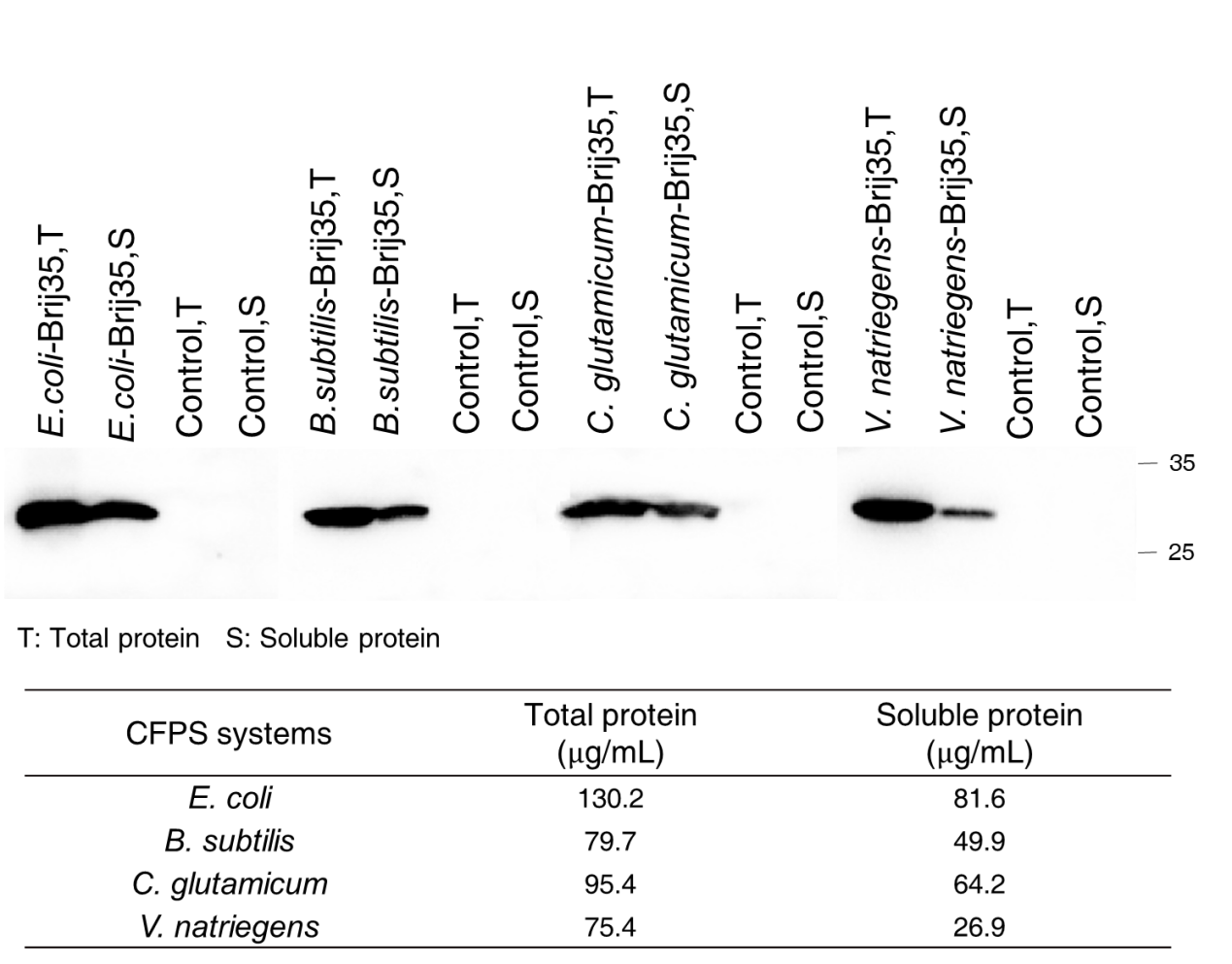


**Fig. S4.** Western blotting and quantitative analysis of RBD-Foldon in *E. coli*, *B. subtilis*, *C. glutamicum* and *V. natriegens* CFPS systems.


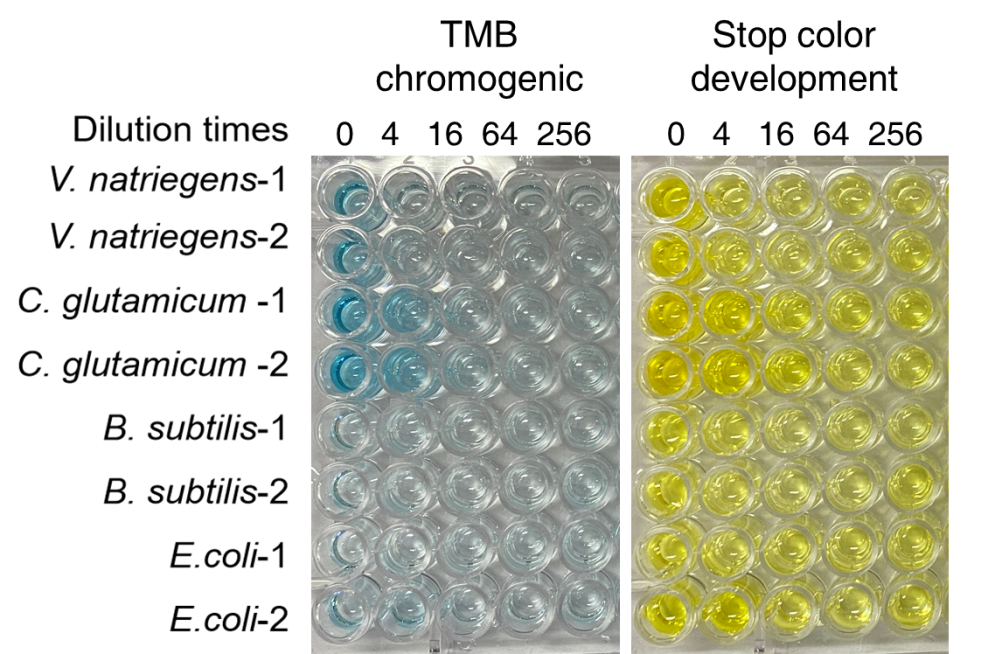


**Fig. S5.** Color change of four CFPS samples after TMB development and after termination of color development.


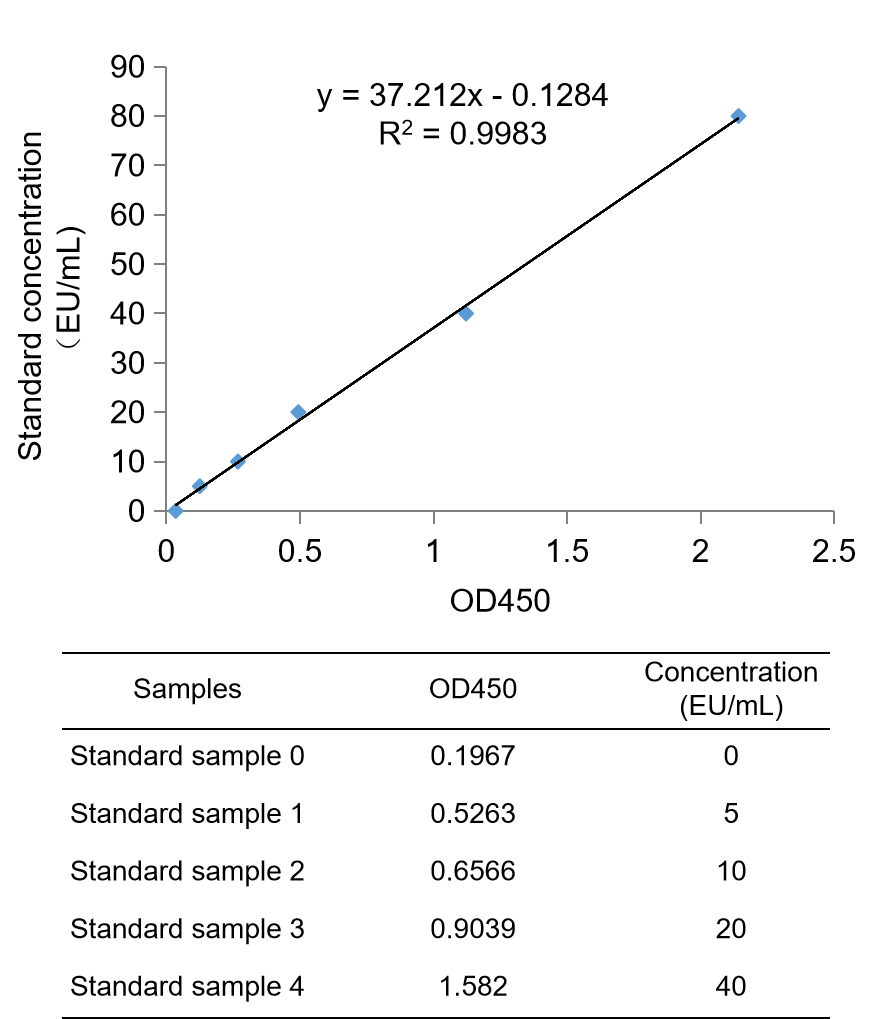


**Fig. S6.** The standard curve of endotoxin.


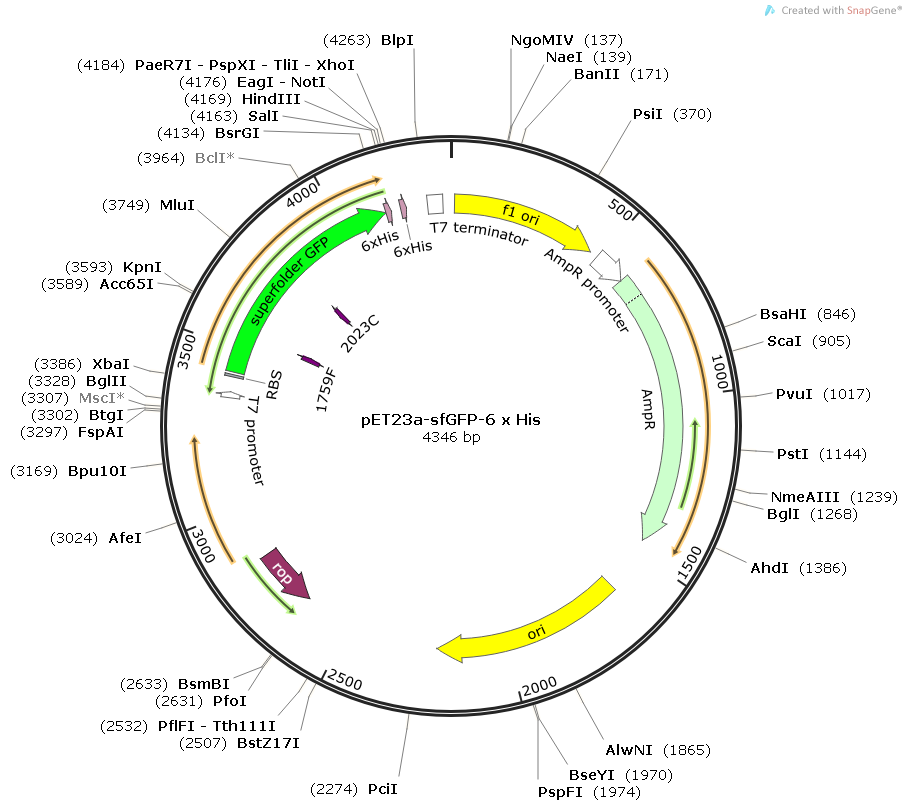


**Fig. S7.** The map of pET23a-sfGFP-6×His.


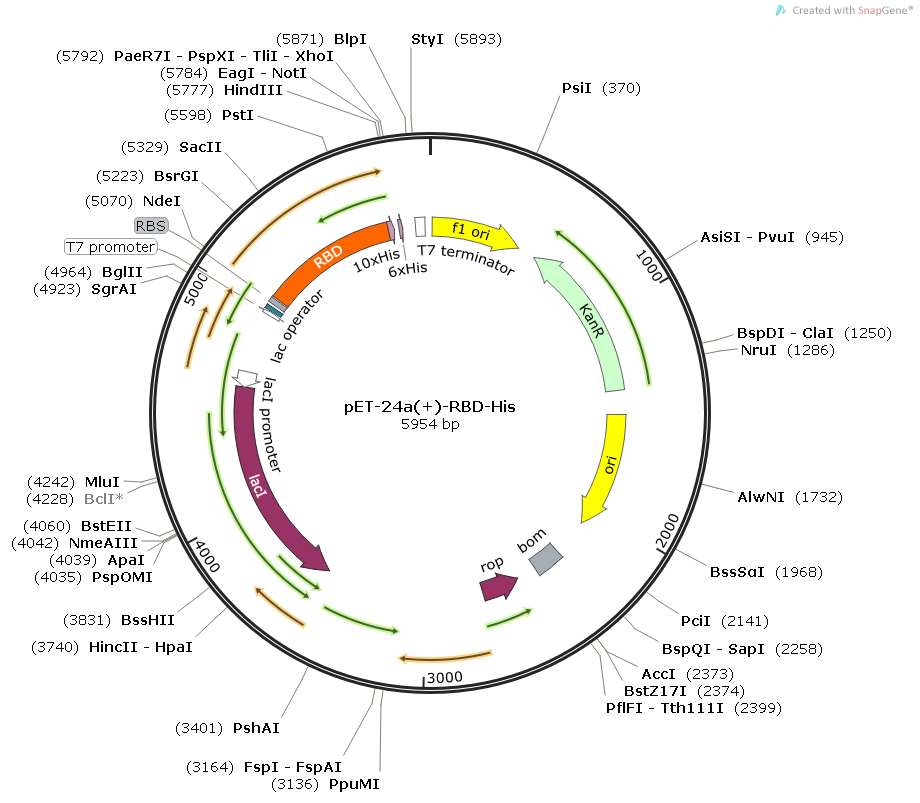


**Fig. S8.** The map of pET24a (＋)-RBD-His.

**
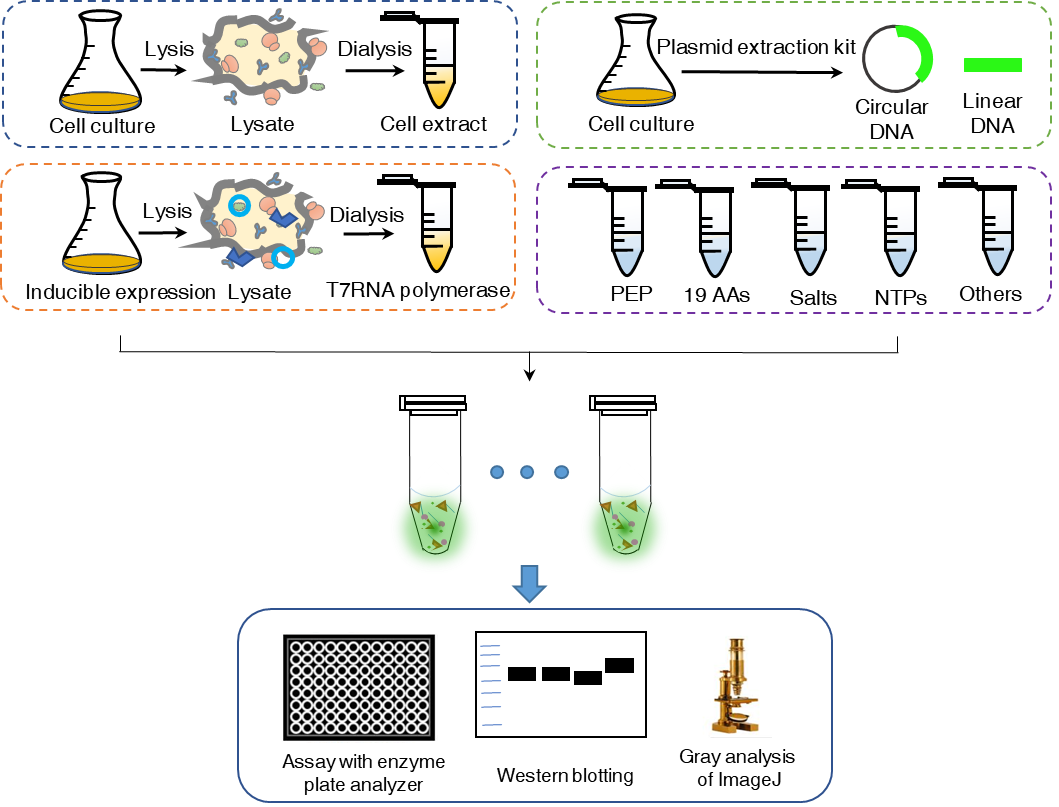
**

**Fig. S9.** Flow chart of experimental design.

**Table S1** The strains in this study

| Strains | Characteristics | Source |
| --- | --- | --- |
| *C. glutamicum* MB001 (DE3) | MB001 derivative with chromosomally encoded T7 gene *1* (cg1122‐P*lacI‐lacI*‐P*lac*UV5–*lacZ*α‐T7 gene *1*‐cg1121) | Gifted from Chen Zhen Research Group (Kortmann et al. 2015) |
| *V. natriegens* Vmax | Vmax™ Express containing a major extracellular nuclease knockout and insertion of an IPTG-inducible T7 RNA polymerase cassette for expression of genes under a T7 promoter | Gifted from Chen Zhen Research Group (Des Soye et al. 2018) |
| *B. subtilis* 168 | trpC2 | Buy from the Guangdong Industrial Microorganism Storage Bank (Kelwick et al. 2016) |
| *E. coli* BL21 star (DE3) | rne131 mutant, enhancing the stability of intracellular mRNA | (Jiang et al. 2021) |

**Table S2** The concentrations of components in original reference CFPS systems

| Component | Concentration | Company |
| --- | --- | --- |
| Potassium glutamate | 175 mM | Vetec (Sigma-Aldrich), Shanghai, China |
| Ammonium glutamate | 10 mM | Vetec (Sigma-Aldrich), Shanghai, China |
| Sodium oxalate | 2.7 mM | Vetec (Sigma-Aldrich), Shanghai, China |
| Spermidine | 1.5 mM | Vetec (Sigma-Aldrich), Shanghai, China |
| Putrescine | 1.0 mM | Vetec (Sigma-Aldrich), Shanghai, China |
| Natural amino acids | 2 mM | Vetec (Sigma-Aldrich), Shanghai, China |
| Magnesium glutamate | 20 mM | Vetec (Sigma-Aldrich), Shanghai, China |
| Glutathione disulfide (GSSG) | 4 mM | Solarbio, Beijing, China |
| Glutathione (GSH) | 1 mM | Solarbio, Beijing, China |
| β-NAD | 0.33 mM | Vetec (Sigma-Aldrich), Shanghai, China |
| CoA | 0.27 mM | Sigma, Co.St.Louis, MO, USA |
| ATP | 1.2 mM | Sangon, Shanghai, China |
| GMP, UMP, CMP | 0.86 mM | Sangon, Shanghai, China |
| Phosphoenolpyruvate (PEP) | 33 mM | Alfa Aesar, Shanghai, China |
| Folinic acid | 34 μg/mL | Aladdin, Shanghai, China |
| E. coli tRNA mixture | 170 μg/mL | Roche, Indianapolis, IN, USA |
| DNA template (plasmid) | 300 ng/μL | Preparation methods in reference study |
| T7 RNA polymerase | 0.2 μL | Preparation methods in reference study |
| Cell extract | 5 μL | Preparation methods in reference study |
|  |  |  |

**Table S3** Addition gradient of CFPS system components

| The gradient | A | B | C | D | E | F |
| --- | --- | --- | --- | --- | --- | --- |
| 1 | 0 | 0 | 0 | 0 | 0 | 0 |
| 2 | 8 | 20 | 0.2 | 0.5 | 1:8 | 1% |
| 3 | 10 | 25 | 0.4 | 1 | 1:4 | 1.5% |
| 4 | 20 | 33 | 0.8 | 2 | 4:4 | 2% |
| 5 | 40 | 50 | 1.2 | 3 | 4:1 | 2.5% |
| 6 |  | 100 | 1.6 | 4 | 8:1 | 3% |

*A: Mg^2+^ concentrations (mM); B: PEP concentrations (mM); C: The volume of NTPs (µL); D: 19AAs concentrations (mM); E: Oxidation reductant (GSSG,GSH) ratio; F: PEG8000 (Volume ratio).

**Table S4** Optimum conditions in the *V. natriegens*, *C. glutamicum*, *B. subtilis* and *E. coli* CFPS expression systems.

| Systems | Components | Concentration | Components | Concentration |
| --- | --- | --- | --- | --- |
| *V. natriegens* | PEG8000 | 1.5 % | NAD | 0.165 mM |
|  | Potassium glutamate | 175 mM | CoA | 0.135 mM |
|  | Ammonium glutamate | 10 mM | ATP | 0.6 mM |
|  | Sodium oxalate | 2.7 mM | GMP,UMP,CMP | 0.43 mM |
|  | PEP | 20 mM | tRNA | 85 μg/mL |
|  | T7 RNA polymerase | 0.2 μL | Folinic acid | 17 μg/mL |
|  | Natural amino acids | 0.5 mM | Spermidine | 0.75 mM |
|  | Magnesium glutamate | 10 mM | Putrescine | 0.5 mM |
|  | GSSG | 1 mM | DNA template (plasmid) | 600 ng/μL |
|  | GSH | 4 mM | Cell extract | 5 μL |
| *C. glutamicum* | PEG8000 | 1.5 % | NAD | 0.0825 mM |
|  | Potassium glutamate | 175 mM | CoA | 0.0675mM |
|  | Ammonium glutamate | 10 mM | ATP | 0.3 mM |
|  | Sodium oxalate | 2.7 mM | GMP,UMP,CMP | 0.215 mM |
|  | PEP | 20 mM | tRNA | 42.5 μg/mL |
|  | T7 RNA polymerase | 0.2 μL | Folinic acid | 8.5 μg/mL |
|  | Natural amino acids | 2 mM | Spermidine | 0.375 mM |
|  | Magnesium glutamate | 10 mM | Putrescine | 0.25 mM |
|  | GSSG | 1 mM | DNA template (plasmid) | 1200 ng/μL |
|  | GSH | 8 mM | Cell extract | 5 μL |
| *B. subtilis* | PEG8000 | 2.5 % | NAD | 0.165 mM |
|  | Potassium glutamate | 175 mM | CoA | 0.135 mM |
|  | Ammonium glutamate | 10 mM | ATP | 0.6 mM |
|  | Sodium oxalate | 2.7 mM | GMP,UMP,CMP | 0.43 mM |
|  | PEP | 33 mM | tRNA | 85 μg/mL |
|  | T7 RNA polymerase | 0.2 μL | Folinic acid | 17 μg/mL |
|  | Natural amino acids | 3 mM | Spermidine | 0.75 mM |
|  | Magnesium glutamate | 20 mM | Putrescine | 0.5 mM |
|  | GSSG | 1 mM | DNA template (plasmid) | 700 ng/μL |
|  | GSH | 8 mM | Cell extract | 5 μL |
| *E. coli* | PEG8000 | 1.5 % | NAD | 0.165 mM |
|  | Potassium glutamate | 175 mM | CoA | 0.135 mM |
|  | Ammonium glutamate | 10 mM | ATP | 0.6 mM |
|  | Sodium oxalate | 2.7 mM | GMP,UMP,CMP | 0.43 mM |
|  | PEP | 20 mM | tRNA | 85 μg/mL |
|  | T7 RNA polymerase | 0.2 μL | Folinic acid | 17 μg/mL |
|  | Natural amino acids | 4 mM | Spermidine | 0.75 mM |
|  | Magnesium glutamate | 10 mM | Putrescine | 0.5 mM |
|  | GSSG | 4 mM | DNA template (plasmid) | 300 ng/μL |
|  | GSH | 1 mM | Cell extract | 5 μL |

**Table S5** The correlation analysis of different reagent components in four CFPS systems

| Reagent composition | Correlation (r) *** | | | |
| --- | --- | --- | --- | --- |
|  | *V. natriegens* | *B. subtilis* | *C. glutamicum* | *E. coli* |
| Mg^2+^ | -0.25508 | 0.044104 | -0.27596 | 0.357121 |
| PEP | -0.24011 | -0.31227 | -0.36678 | -0.37164 |
| 19 AA | 0.072264 | 0.21845 | -0.19118 | 0.86327 |
| GSSG/GSH | 0.192949 | -0.14529 | -0.08085 | 0.790371 |
| PEG8000 | -0.68266 | 0.040875 | 0.962762 | 0.421173 |
| NTPs | 0.174451 | -0.449 | -0.02421 | 0.205778 |

*Weak correlation: -0.3<r<0.3; Intermediate correlation: -0.6<r<-0.3, 0.3<r<0.6; High correlation: -1<r<-0.6, 0.6<r<1.

**Table S6** The genetic sequences used in this study

| **sfGFP-6×His**  taatacgactcactatagggagaccacaacggtttccctctagaaataattttgtttaactttaagaaggagatatacatATGCGTAAAGGCGAAGAGCTGTTCACTGGTGTCGTCCCTATTCTGGTGGAACTGGATGGTGATGTCAACGGTCATAAGTTTTCCGTGCGTGGCGAGGGTGAAGGTGACGCAACTAATGGTAAACTGACGCTGAAGTTCATCTGTACTACTGGTAAACTGCCGGTACCTTGGCCGACTCTGGTAACGACGCTGACTTATGGTGTTCAGTGCTTTGCTCGTTATCCGGACCATATGAAGCAGCATGACTTCTTCAAGTCCGCCATGCCGGAAGGCTATGTGCAGGAACGCACGATTTCCTTTAAGGATGACGGCACGTACAAAACGCGTGCGGAAGTGAAATTTGAAGGCGATACCCTGGTAAACCGCATTGAGCTGAAAGGCATTGACTTTAAAGAAGACGGCAATATCCTGGGCCATAAGCTGGAATACAATTTTAACAGCCACAATGTTTACATCACCGCCGATAAACAAAAAAATGGCATTAAAGCGAATTTTAAAATTCGCCACAACGTGGAGGATGGCAGCGTGCAGCTGGCTGATCACTACCAGCAAAACACTCCAATCGGTGATGGTCCTGTTCTGCTGCCAGACAATCACTATCTGAGCACGCAAAGCGTTCTGTCTAAAGATCCGAACGAGAAACGCGATCATATGGTTCTGCTGGAGTTCGTAACCGCAGCGGGCATCACGCATGGTATGGATGAACTGTACAAACATCACCATCACCATCATTAAgtcgacaagcttgcggccgcactcgagcaccaccaccaccaccactgagatccggctgctaacaaagcccgaaaggaagctgagttggctgctgccaccgctgagcaataactagcataaccccttggggcctctaaacgggtcttgaggggttttttg |
| --- |
| **sfGFP codon optimization based on *E. coli***  ATGCGTAAAGGTGAAGAACTGTTTACCGGTGTGGTTCCGATTCTGGTTGAACTGGATGGCGATGTTAATGGTCATAAATTTTCAGTGCGCGGCGAAGGTGAAGGCGATGCCACCAATGGCAAACTGACCCTGAAATTCATTTGCACCACCGGTAAACTGCCGGTGCCGTGGCCGACCCTGGTGACAACCCTGACCTATGGCGTGCAGTGTTTTGCCCGCTATCCGGATCATATGAAACAGCATGATTTCTTTAAGAGCGCAATGCCGGAAGGTTATGTGCAGGAACGTACCATTAGTTTTAAAGATGATGGTACCTATAAGACCCGTGCAGAAGTGAAATTTGAAGGTGACACCCTGGTGAATCGCATTGAACTGAAAGGCATTGATTTTAAAGAGGATGGTAATATTCTGGGCCATAAACTGGAATATAATTTTAATAGCCACAACGTTTACATCACCGCCGATAAACAGAAAAATGGTATTAAGGCAAACTTCAAAATCCGTCATAATGTTGAAGATGGTAGCGTGCAGCTGGCAGATCATTATCAGCAGAATACCCCGATTGGTGACGGTCCGGTTCTGCTGCCGGATAATCATTATCTGAGTACCCAGAGTGTTCTGAGCAAAGATCCGAATGAAAAACGTGATCATATGGTTCTGCTGGAATTTGTTACCGCCGCAGGCATTACCCATGGTATGGATGAACTGTATAAA |
| **sfGFP codon optimization based on** ***B. subtilis*** ATGCGCAAAGGCGAAGAACTGTTCACGGGCGTCGTCCCGATTCTTGTTGAACTTGACGGCGACGTTAACGGCCACAAATTCAGCGTCAGAGGCGAAGGCGAAGGAGACGCCACAAATGGCAAGCTGACGCTGAAGTTCATCTGCACGACGGGCAAACTGCCGGTTCCGTGGCCGACGCTTGTCACGACGCTGACATATGGCGTCCAGTGTTTTGCTCGCTACCCGGACCACATGAAGCAGCACGACTTCTTCAAGAGCGCCATGCCGGAAGGCTACGTCCAAGAACGCACGATCTCCTTCAAGGACGACGGCACGTACAAAACGCGCGCCGAGGTCAAATTCGAGGGAGACACGCTGGTTAACCGCATCGAGCTGAAGGGCATCGACTTCAAAGAGGACGGCAACATTCTTGGCCACAAGCTGGAGTACAACTTCAACAGCCACAATGTCTACATCACGGCCGACAAGCAGAAGAACGGCATCAAGGCCAACTTCAAGATCCGCCACAACGTCGAAGATGGCAGCGTCCAGCTTGCCGATCACTACCAGCAGAACACACCGATCGGCGATGGCCCGGTCCTTCTTCCGGACAATCACTATCTTTCCACACAGTCCGTTCTTTCCAAAGACCCGAACGAGAAACGCGACCATATGGTTCTTCTGGAGTTTGTCACGGCTGCCGGCATCACGCATGGCATGGATGAGCTGTACAAGCACCACCACCATCATCACTAA |
| **sfGFP codon optimization based on *V. Natriegens* and *C. glutamicum*** ATGCGTAAGGGCGAGGAACTGTTTACCGGCGTCGTGCCAATTCTGGTGGAGCTGGACGGCGACGTCAACGGCCACAAGTTTTCCGTGCGCGGTGAAGGCGAAGGCGACGCTACCAACGGCAAGCTGACTCTGAAGTTCATCTGTACCACCGGCAAGCTGCCAGTGCCTTGGCCAACTCTGGTCACCACCCTCACCTACGGCGTGCAGTGTTTCGCTCGCTACCCAGATCACATGAAGCAGCACGACTTTTTCAAGTCCGCTATGCCAGAGGGCTACGTGCAAGAACGCACCATCTCCTTCAAGGACGACGGCACCTACAAGACCCGTGCAGAGGTGAAGTTCGAGGGCGATACTCTGGTCAACCGTATCGAGCTGAAGGGCATCGACTTCAAGGAAGACGGCAACATTCTGGGTCACAAGCTGGAGTACAACTTCAACTCCCACAACGTCTACATCACCGCTGACAAGCAGAAGAACGGCATCAAGGCTAACTTCAAGATCCGCCACAACGTGGAGGACGGTTCCGTCCAGCTGGCAGATCACTACCAGCAGAACACCCCAATCGGCGACGGTCCAGTGCTGCTGCCAGACAACCACTATCTGTCCACCCAGTCTGTGCTGTCCAAGGATCCAAACGAGAAGCGCGATCACATGGTGCTGCTGGAGTTCGTGACCGCAGCTGGCATCACCCATGGCATGGATGAGCTGTACAAGCACCACCACCACCATCACTAA |
| **RBD-Foldon-His**  taatacgactcactataggggaattgtgagcggataacaattcccctctagaaataattttgtttaactttaagaaggagatataCATATGCGCGTTCAGCCTACAGAATCAATTGTTCGCTTTCCTAACATTACAAACCTTTGTCCTTTCGGCGAAGTCTTCAATGCGACACGCTTTGCTTCAGTTTATGCTTGGAACCGCAAACGCATTTCAAACTGTGTTGCTGATTATTCAGTTCTTTATAACTCAGCTTCATTCTCGACGTTTAAATGTTATGGCGTTTCACCTACAAAGCTAAATGATCTTTGTTTCACTAATGTTTATGCTGATTCATTTGTTATTCGCGGCGATGAAGTTCGCCAGATTGCTCCTGGCCAGACAGGCAAGATAGCGGATTATAACTATAAACTTCCTGATGATTTCACGGGATGTGTTATTGCTTGGAACTCAAACAACCTTGATTCAAAGGTGGGTGGTAATTATAACTATCTTTATCGCCTGTTCCGGAAGTCAAACCTTAAACCTTTCGAGAGAGATATTTCAACAGAAATTTATCAGGCTGGCTCAACACCTTGTAACGGCGTTGAAGGCTTTAACTGTTATTTCCCACTGCAGTCTTATGGCTTTCAGCCTACAAACGGCGTTGGCTATCAGCCTTATCGCGTTGTTGTTCTTTCATTTGAACTTCTTCATGCTCCTGCTACAGTTTGTGGCCCTAAGAAGTCGACCAACCTTGTTAAGAATAAGTGTGTTAACTTTGGCTCAGGCTATATTCCTGAAGCTCCTCGCGATGGCCAGGCTTATGTTCGCAAAGATGGCGAATGGGTTCTTCTTTCAACATTTCTTGGCCATCACCACCATCATCATCACCATCACCATTAAAAGCTTgcggccgcactcgagcaccaccaccaccaccactgagatccggctgctaacaaagcccgaaaggaagctgagttggctgctgccaccgctgagcaataactagcataaccccttggggcctctaaacgggtcttgaggggttttttg |

**References**

Kortmann M, Kuhl V, Klaffl S et al (2015) *A chromosomally encoded T7 RNA polymerase-dependent gene expression system for Corynebacterium glutamicum: construction and comparative evaluation at the single-cell level*. *Microbial biotechnology* **8**:253-265.

Des Soye BJ, Davidson SR, Weinstock MT et al (2018) *Establishing a High-Yielding Cell-Free Protein Synthesis Platform Derived from Vibrio natriegens*. *ACS Synth Biol* **7**:2245-2255.

Kelwick R, Webb AJ, MacDonald JT et al (2016) *Development of a Bacillus subtilis cell-free transcription-translation system for prototyping regulatory elements*. *Metabolic engineering* **38**:370-381.

Jiang N, Ding X, Lu Y (2021) *Development of a robust Escherichia coli-based cell-free protein synthesis application platform*. *Biochemical engineering journal* **165**:107830.
